# Protein phosphatase 2A impairs cardiac uptake of glucose in the mammalian heart

**DOI:** 10.1007/s00210-026-05617-x

**Published:** 2026-06-25

**Authors:** Katarina Hadova, Daniel Gündel, Torsten Knieß, Peter Brust, Jan Klimas, Joachim Neumann, Ulrich Gergs

**Affiliations:** 1 Faculty of Pharmacy, Comenius University in Bratislava, Odbojarov Street 10, Bratislava 3, 832 32 Slovak Republic; 2 Department of Neuroradiopharmaceuticals, Helmholtz-Zentrum Dresden-Rossendorf, Institute of Radiopharmaceutical Cancer Research, Research Site Leipzig, Permoser Straße 15, 04813 Leipzig, Germany; 3 Helmholtz-Zentrum Dresden-Rossendorf, Institute of Radiopharmaceutical Cancer Research, Bautzner Landstraße 400, 01328 Dresden, Germany; 4 University Medical Center Schleswig-Holstein, The Lübeck Institute of Experimental Dermatology, Lübeck, Germany; 5 Institut für Pharmakologie und Toxikologie, Medizinische Fakultät, Martin-Luther-Universität Halle-Wittenberg, Magdeburger Straße 4, Halle, 06097 Germany

**Keywords:** Protein phosphatase 2A, Glucose metabolism, Heart, Transgenic mice, 2-[^18^F]fluoro- 2-deoxy-D-glucose ([^18^F]FDG)

## Abstract

Protein phosphatase 2A (PP2A) accounts for approximately half of all serine/threonine phosphatase activity in the mammalian heart. We hypothesised that PP2A, through dephosphorylation of key regulatory enzymes, modulates cardiac glucose uptake and metabolism in vivo. Using positron emission tomography (PET), we studied the uptake of 2-[^18^F]fluoro-2-deoxy-D-glucose ([^18^F]FDG) in living mice with cardiac-specific overexpression of the catalytic subunit of PP2A (PP2A-TG) as well as in littermate controls (wild-type, WT). Additionally, we analysed the expression of enzymes and transcription factors involved in cardiac glucose metabolism. Cardiac glucose consumption was reduced by 41% (*P* < 0.05) in PP2A-TG mice relative to WT. Real-time quantitative polymerase chain reaction analysis revealed decreased gene expression of glycogen synthase 1 (− 26%, *P* < 0.05), glycogen phosphorylase (− 17%, *P* < 0.05), Mtor (-22%, *P* < 0.05), peroxisome proliferator-activated receptor gamma (Pparg), and its coactivator PGC-1α (-41% and − 28% respectively; both *P* < 0.05) in PP2A-TG hearts. Western blotting demonstrated increased protein expression of phosphoinositide 3-kinase (PI3K, 26%, *P* < 0.05), reduced expression of glycogen synthase (-34%, *P* < 0.05) and glycogen phosphorylase (-18%, *P* < 0.05), and enhanced phosphorylation of AKT (95%, *P* < 0.05) and glycogen synthase kinase-3β (GSK3β, 22%, *P* < 0.05) in PP2A-TG hearts. These findings indicate that PP2A impairs cardiac glucose metabolism in vivo through modulation of multiple components of glucose handling, including the PI3K/AKT signalling axis, glycogen metabolism, and PPARγ-dependent transcriptional regulation. The effects of PP2A appear to be predominantly indirect. Elevated PP2A expression may therefore be detrimental in heart failure by compromising myocardial energy production.

**Supplementary Information:**

The online version contains supplementary material available at https://doi.org/10.1007/s00210-026-05617-x.

## Introduction

Reversible protein phosphorylation is one of the essential regulatory mechanisms governing numerous key cellular functions. In the mammalian heart, more than 90% of total serine/threonine phosphatase activity is mediated by four major phosphatases, including protein phosphatase 2 A (PP2A) (Heijman et al. 2013; Neumann et al. 2021). PP2A is considered a central regulator of multiple cellular functions in both healthy and diseased myocardium (Bokník et al. 2000; Abdallah et al. 2022). Experiments with transgenic mice with cardiac-specific overexpression of PP2A catalytic subunit α showed impairments in cardiac structure and function, such as cardiac hypertrophy, reduced contractility of the heart and cardiac dilatation, without an increase in morbidity and mortality, however. Moreover, these animals exhibited decreased expression and/or phosphorylation of some excitation-contraction-coupling proteins and the presence of necrotic isles within the myocardium, and they developed left ventricular fibrosis (Gergs et al. 2004; Hoehn et al. 2015).

Accumulating evidence suggests that PP2A is involved in glucose handling and metabolism. Although glucose is not the primary substrate for ATP production in the healthy adult heart, it becomes a critical energy source under pathological conditions, such as myocardial ischemia and infarction, where oxygen availability is limited and anaerobic glycolysis predominates (Stanley et al. 2005; Chen et al. 2022). First, PP2A is implicated in the regulation of myocardial glucose uptake. PP2A regulates insulin signalling via the PI3K/AKT pathway (Ugi et al. 2004; Bernsmeier et al. 2008; Xiang et al. 2025), which is crucial for insulin-stimulated translocation of glucose transporter 4 (GLUT4) to the cell membrane and consequently glucose uptake (Fazakerley et al. 2022). Second, PP2A might be involved in the regulation of glycolysis through modulation of the hexokinases activity (Lama et al. 2013). As hexokinases represent the first rate-limiting step in glucose utilisation (De Jesus et al. 2022), PP2A could substantially affect the key step in glycolysis. Third, PP2A indirectly contributes to glycogen metabolism in the heart (Sergienko et al. 2022). Cardiac glycogen is utilised by the aerobic working muscle and is even preferentially oxidised compared to exogenous glucose (Pederson et al. 2004). The release of glucose from the glycogen macromolecule is in fact regulated by PP2A through dephosphorylation of the enzyme phosphorylase, resulting in the suppression of glycogen breakdown (Bollen et al. 1998). Taken together, these observations suggest that PP2A may exert multiple regulatory effects on cardiac glucose metabolism; however, its precise role in myocardial glucose handling in vivo needs to be addressed.

Hence, we tested the hypothesis that mice with enhanced expression and activity of PP2A show altered glucose handling in the heart. Furthermore, we aimed to elucidate the underlying mechanism(s) for these alterations in glucose metabolism.

## Materials and methods

### Transgenic mice

Transgenic mice were obtained from an established founder lineage generated as described previously (Gergs et al. 2004). Briefly, cDNA encoding mouse PP2Acα was generated using the reverse transcription-polymerase chain reaction methodology (flanked with NotI and SalI sites). The NotI-SalI restriction enzyme fragment was ligated into the same sites of a mouse cardiac α-myosin heavy chain promoter expression cassette containing the SV40 transcriptional terminator. The transgene was isolated from the parent plasmid as a 7-kilobase NruI fragment and used for the microinjection of fertilised mouse eggs (CD1). Transgene-positive mice were identified by Southern blotting and PCR assay of tail genomic DNA.

All research procedures followed the NIH Guide for the Care and Use of Laboratory Animals (NIH publication number 85–23, revised 2011). Adult PP2A transgenic mice** (**PP2A-TG) 7–11 months old and wild type (WT) littermates with a body weight of at least 35 g were used. All animals had free access to water and the Altromin 1320 ® standard diet. All diagnostic procedures were carried out with the investigator blinded in regard to the genotype of the mice and treatment group.

### mRNA extraction and quantification

The mice were euthanised and retrogradely perfused with Tyrode’s solution in order to wash out the blood. Thereafter, the hearts were rapidly frozen in liquid nitrogen and homogenised in a frozen state. The homogenates were kept at °−80 °C until biochemical analysis.

RNA was extracted by acid phenol–guanidinium thiocyanate–chloroform extraction (TRI Reagent®, Sigma- Aldrich, St. Louis, MO, USA) according to the instructions of the manufacturer. The quality of the RNA was verified by agarose gel electrophoresis. Reverse transcription of intact RNA was performed using the High Capacity cDNA Reverse Transcription Kit with RNAse inhibitors (Applied Biosystems, Grand Island, NY, USA). Quantitative real-time PCR was performed on a QuantStudio™ 3 Real-Time PCR System (Thermo Fisher Scientific, USA) using the SYBR™ Select Master Mix (Thermo Fisher Scientific, USA). First, we verified the expression of the exogenous gene construct of PP2A in transgenic animals using primers specific to the exogenous gene construct. We subsequently assessed the expression of the genes involved in glucose metabolism. The list of analysed genes and the specific primers designed using Primer-BLAST (Ye et al. 2012) are shown in Table 1. The resulting relative expression was calculated according to the Pfaffl method (Pfaffl 2001). The results were normalised to the reference gene hypoxanthine phosphoribosyltransferase 1 (Hprt1) and calibrated to the wild type group.

**Table 1:** Primer sequences for RT-qPCR

*Primer sequences for RT-qPCR*			
Gene	RefSeq accession number	Primer sequence (5´−3´)	PCR product length (bp)
Akt2	NM_001331108.1	Forward: TGCCACCCTTCAAACCTCAG Reverse: GGGTCCAGGCTGTCATATCG	115
Gsk3b	NM_019827.7	Forward: TTATCCCTCCACATGCTCGG Reverse: CGGTCTCCAGCATTAGTATCTGA	85
Gys1	NM_030678.3	Forward: GCTGGCCGCTATGAGTTTTC Reverse: GCTCACTGCCATTCACTCTG	94
Hk1	NM_001146100.1	Forward: CCGCCATTGAAACGGATAAGG Reverse: GCATACGTGCTGGACCGATA	107
Hk2	NM_013820.4	Forward: GGCTAGGAGCTACCACACAC Reverse: AACTCGCCATGTTCTGTCCC	91
Hprt1	NM_013556.2	Forward: TTGGGCTTACCTCACTGCTT Reverse: ATCATCGCTAATCACGACGC	132
Insr	NM_001330056.1	Forward: GCCAGTCTTCGAGAACGGAT Reverse: GCCAGTCTTCGAGAACGGAT	85
Irs1	NM_010570.4	Forward: ATGAGGGCAACTCCCCAAGA Reverse: AGGTCATTTAGGTCTTCATTCTGCT	130
Mtor	NM_020009.2	Forward: GGAGGTGTGGTTTGACCGAA Reverse: GATTGGATGGGTGCCTGTCT	101
Pik3ca	NM_008839.3	Forward: CGAAGTGGCCCAGATGTACT Reverse: GCACCGAACAGCAAAACTCC	121
Pparg	NM_001308352.2	Forward: GCTCCAAGAATACCAAAGTGCG Reverse: ACAGACTCGGCACTCAATGG	133
Ppargc1a	NM_008904.3	Forward: CCCTGCCATTGTTAAGACCG Reverse: GGAGGGTCATCGTTTGTGGT	126
Prkaa2	NM_178143.2	Forward: AGGTCATCTCAGGAAGGCTGTA Reverse: GTAAAACACACCCCCTCGGA	149
Ppp2ca	NM_019411.4	Forward: AGAGTTTGAGTGACCGCGTCT Reverse: CGTTCAGCTGCTCGATCCAC	150
Pygb	NM_153781.1	Forward: TACTTCTTCGCTCTGGCACA Reverse: GGTAATAGATGCGCTTGGGG	103
Pygm	NM_011224.2	Forward: CATCAACCCCAACTCGCTCT Reverse: GGCTCCCTTTTGATGCGGTT	114
Slc2a1	NM_011400.3	Forward: GCTGTCGGGTATCAATGCTG Reverse: GCTCTACAACAAACAGCGACA	140
Slc2a4	NM_001359114.1	Forward: CTGGTTCATTGTGGCAGAGC Reverse: AAGGACCCATAGCATCCGCA	134

*Akt2* Akt serine/threonine kinase 2, *Gsk3b* glycogen synthase kinase 3 beta, *Gys1* glycogen synthase 1, *Hk1* hexokinase 1, *Hk2* hexokinase 2, *Hprt1* hypoxanthine guanine phosphoribosyl transferase, *Insr* insulin receptor, *Irs1* insulin receptor substrate 1, *Mtor* mechanistic target of rapamycin kinase, *Pik3ca* phosphatidylinositol-4,5-bisphosphate 3-kinase catalytic subunit alpha (alias PI3K), *Pparg* peroxisome proliferator activated receptor gamma, *Ppargc1a* PPARG coactivator 1 alpha, *Prkaa2* protein kinase (alias PGC-1α), AMP-activated (alias AMPK), alpha 2 catalytic subunit, *Ppp2ca* protein phosphatase 2 (formerly 2A), catalytic subunit, alpha isoform, *Pygb* brain glycogen phosphorylase, *Pygm* muscle glycogen phosphorylase, *Slc2a1* solute carrier family 2 member 1 (alias Glut1), *Slc2a4* solute carrier family 2 member 4 (alias Glut4)

### Positron Emission Tomography (PET)

[^18^F]−2-fluoro-2-deoxy-D-glucose ([^18^F]FDG) PET imaging was performed to measure the myocardial glucose consumption. Between 6 and 12 MBq [^18^F]FDG were injected i.p. into WT (*n* = 5, 3 male, 2 females, weighing 30 to 40 g) and PP2A-TG (*n* = 8, 3 male, 5 female, weighing 34 to 47 g) mice, respectively. The animals were injected in the awake state and remained in their home cages after injection. After 30 min, the animals were anaesthetised with 2% isoflurane in 60% oxygen and placed on a thermostatically-heated animal bed. PET scanning in a small-animal PET/MR (Nanoscan, Mediso, Budapest, HUN) started 45 min after [^18^F]FDG injection. Static PET scans were performed, followed by T1-weighted GRE MR imaging for anatomical correlations and attenuation correction. PET acquisition time was 15 min. List mode data from the PET scans were reconstructed by Ordered Subset Expectation Maximisation (OSEM3D) with four iterations, six subsets and a voxel size of 0.4 mm isotropic 291 (Nucline v2.01, Mediso, HUN). Blood glucose concentration (6.7 to 8.6 mmol/L in WT and 5.4 to 8.3 mmol/L in PP2A-TG, 292 Accu-Chek Performa Nano, Roche, Mannheim, gradient echo magnetic resonance, Germany) were measured shortly before injecting the radiotracer. The analysis of reconstructed datasets was performed with the PMOD software (v4.205, PMOD Technologies LLC, Zurich, Switzerland), and Microsoft Excel was used for statistical analysis (one-sided Student’s *t*-test) of the obtained standardised uptake values (SUVs). The blood glucose corrected SUV_gluc_ was determined according to Wanek et al. (Wanek et al. 2025) and calculated as:

$$SU{V}_{gluc} = SUV\times {c}_{gluc}/100$$

where c_gluc_ is the blood glucose concentration in mg/dL (1 mmol/L ≙ 18.02 mg/dL) measured after PET acquisition.

### Glycogen measurement

To assess the glycogen content in the heart samples, a colorimetric assay using the EnzyChrom™ Glycogen Assay Kit was performed. The samples were first homogenised in citrate buffer (0.1 mol/l, pH 4.2) with NaF addition (250 mg/dl), and the assay was subsequently performed according to the manufacturer’s instructions. The assay combines the enzymatic breakdown of glycogen and the detection of glucose. The colour intensity of the reaction product is directly proportional to the glycogen concentration in the sample. The errors caused by the presence of glucose within the samples are simply eliminated by preparation of a sample blank with an adjusted working reagent.

### Western-blot analysis

Frozen samples of heart ventricles were homogenised in liquid nitrogen and lysed in Tris–HCl buffer containing protease and phosphatase inhibitors. The concentration of proteins was measured using the Pierce™ BCA Protein Assay Kit. Protein lysates, adjusted to the same concentration, were solubilised and denatured in Laemmli sample buffer at 95 °C for 10 min and subsequently subjected to sodium dodecyl sulphate- polyacrylamide gel electrophoresis on 8–12% gels using PowerPac™ HC High-Current Power Supply (Bio-Rad Laboratories, USA) with Tris–Glycine-SDS-based Running buffer. After separation, the proteins were transferred to a polyvinylidene fluoride membrane (Immobilon P®, Millipore Corporation, USA). The membranes were blocked with 5% non-fat dried milk or 5% BSA in TBST and subsequently incubated with primary antibodies diluted in an appropriate buffer solution (for details, see the Drugs and Materials section). Subsequently, the membranes were incubated with the appropriate HRP-conjugated secondary antibodies. Signals were detected using enhanced chemiluminescence (Immobilon Crescendo Western HRP substrate, Millipore) by a chemiluminescence imaging system (UVITEC Imaging Systems, Nine Alliance, Cambridge, United Kingdom). After detection the phosphorylated protein forms were stripped and corresponding unphosphorylated forms were subsequently assessed on the same membrane. Quantification of the band’s densities was performed using densitometry in UVITEC Imaging Systems software (Nine Alliance, Cambridge, United Kingdom). These arbitrary density levels were normalised to the density of reference protein GAPDH (37 kDa), with stable protein expression in the whole set of samples.

### Data analysis

Data are reported as the mean and standard deviation (SD). According to distribution, data were analysed using the unpaired t-test or the Mann–Whitney test. The normality of data distribution was evaluated using the Shapiro–Wilk test. *P* < 0.05 was considered statistically significant. The data were handled by GraphPad Prism 6 for Windows (Graph Pad Software, Inc., version 6.00).

### Drugs and materials

All primers were purchased from Sigma-Aldrich (Saint Louis, MO, USA). The High-Capacity cDNA Reverse Transcription Kit with RNAse inhibitors was purchased from Applied Biosystems (Grand Island, NY, USA). The SYBR™ Select Master Mix and the Pierce™ BCA Protein Assay Kit were purchased from Thermo Fisher Scientific, USA. The EnzyChrom™ Glycogen Assay Kit was purchased from BioAssay Systems (Hayward, CA, USA). The primary antibodies used to detect protein expression were obtained as follows: anti-Akt (#BD610861, BD Transduction Laboratories™), anti-Phospho-Akt (Ser473) (#9271, Cell Signaling Technology), anti-GAPDH (#G9295, Sigma Aldrich), anti-GLUT1 (#ab32551 abcam), anti-GLUT4 (#ab654, abcam), anti-GSK-3 beta (D5C5Z) (#12,456, Cell Signaling Technology), anti-Phospho-GSK-3 beta (Ser9) (5B3) (#9323, Cell Signaling Technology), anti-Glycogen Synthase (GYS1/GYS2) (15B1) (#3886, Cell Signaling Technology), anti-Insulin Receptor beta (4B8) (#3025, Cell Signaling Technology), anti-PI3 Kinase Class III (D4E2) (#3358, Cell Signaling Technology), anti-PYGM (#MA#5-27,442, Invitrogen). The HRP-conjugated secondary antibodies used in the Western blot experiments were goat-anti-rabbit IgG (#A6154, Sigma-Aldrich) and rabbit-anti-mouse IgG (P0260, Dako).

## Results

### PP2A-TG animals overexpress the catalytic subunit α in the heart

**Fig. 1:**
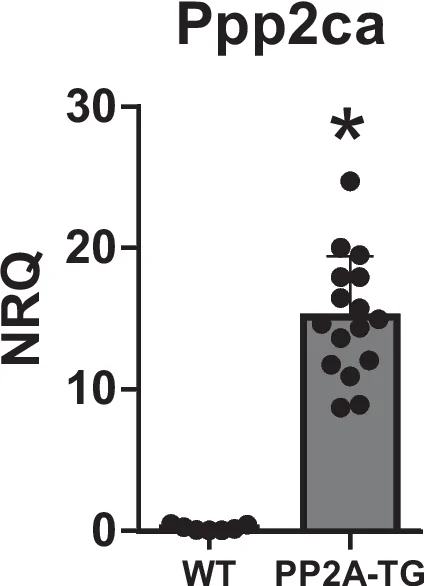
Bar diagram showing gene expression (ordinate) of PP2A (Ppp2ca gene) in the mouse ventricles of PP2A-TG (*n* = 16) and WT (*n* = 7). Data are presented as NRQ ± SD; *P < 0.05 vs WT, (NRQ; normalized relative quantity). Groups labelling: WT – wild type mice, PP2A-TG – mice with overexpression of the catalytic subunit α of protein phosphatase 2A

We confirmed the genotype of the transgenic mice by measuring the transgene expression in the heart of the animals. Since we used the murine Ppp2ca gene, which is expressed in the wild type mice as well, we compared the expression of the Ppp2ca gene in both murine lines and found a marked expression increase (more than 80-fold, *P* < 0.05, *n* = 6–16; Fig. 1) in PP2A-TG compared to WT, which is in line with our previous publication (Gergs et al. 2004).

### Reduced myocardial glucose consumption in PP2A-TG animals

**Fig. 2:**
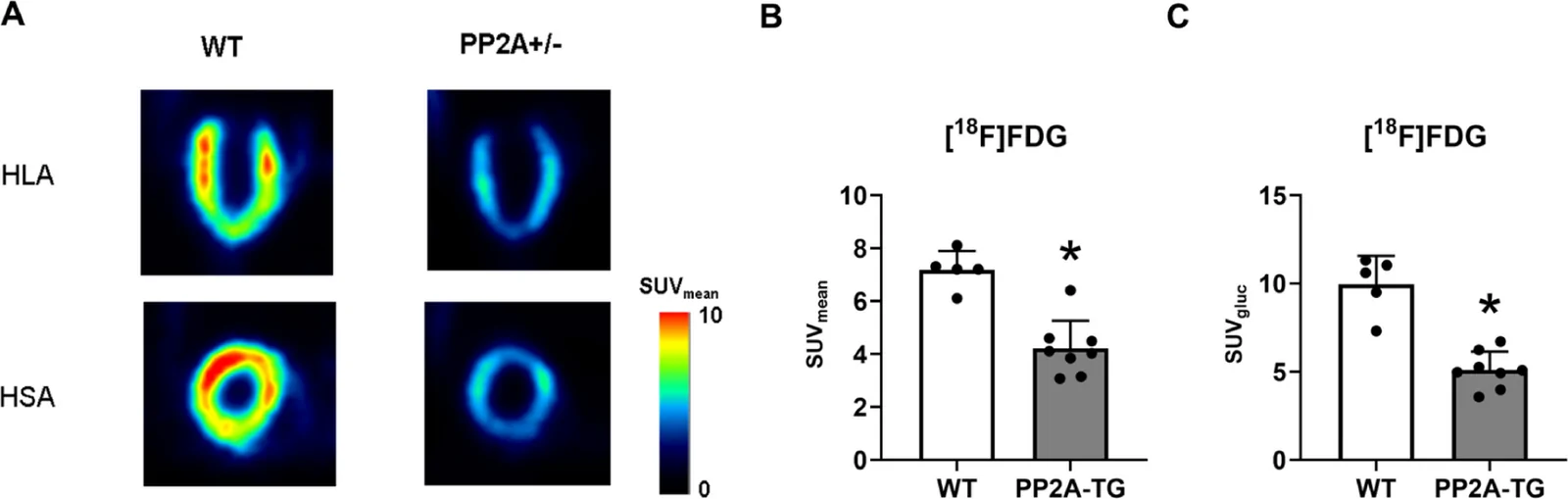
PET. Uptake of [^18^F]FDG into the myocardium of WT and PP2A.^±^. **A** Exemplary images of a static PET of the long axis (HLA) and short axis (HSA) of the heart 45 min after intraperitoneal radiotracer injection (6.2 to 12.0 MBq). **B** Activity concentration in standardised uptake values (SUV ± SD). **C** SUV_gluc_ (https://doi.org/10.1016/j.nucmedbio.2025.109095) in myocardium of WT (*n* = 5) and PP2A ± (*n* = 8)

To determine the effect of PP2A on glucose consumption in the myocardium, we used static [^18^F]FDG PET in anaesthetised animals. A clear signal reduction in PP2A-TG myocardia was visible when comparing the [^18^F]FDG uptake in both models (Fig. 2A). We determined a reduced myocardial glucose consumption by −41 ± 15% (SUV = 4.2 ± 1.0 in TG vs. 7.2 ± 0.7 in WT, *P* < 0.05, *n* = 5–8) in PP2A-TG compared to WT mice (Fig. 2B). The blood glucose-corrected [^18^F]FDG uptake in myocardium was in a similar range, showing a reduction by −48.8% (SUV_gluc_ = 10.0 ± 1.6 in TG vs. 5.1 ± 1.0 in WT, *P* < 0.05, *n* = 5–8) in PP2A-TG compared to WT mice (Fig. 2C). The result indicates a significant role of PP2A in cardiac glucose metabolism.

### PP2A affects insulin receptor pathway

**Fig. 3:**
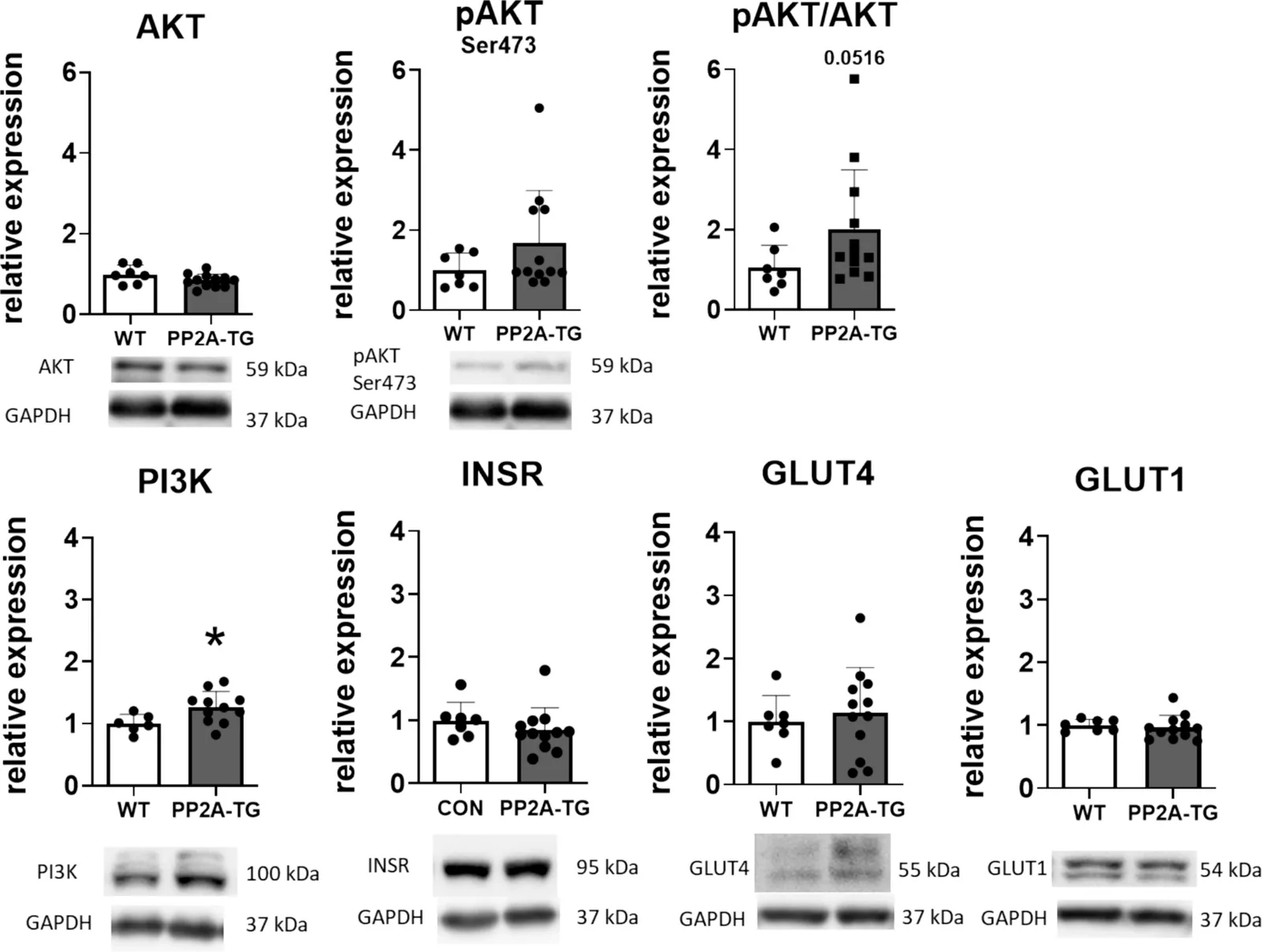
Western blot analysis of proteins of the insulin receptor downstream pathway in the heart of WT and PP2A-TG mice with representative immunoblots. AKT phosphorylation was calculated as the pAKT and AKT expression ratio. The resulting value 0.1 > *P* > 0.05 is a statistical trend and is considered as a non-significant change. Data are presented as mean ± SD; *n* = 7–12 per group; *P < 0.05 vs CON. For groups labelling, see Fig. 1. Full-length blots are presented in Supplementary Figs. 1,2,5 and 6

Glucose can enter cardiomyocytes through activation of insulin receptor and its intrinsic pathway, leading to GLUT4 translocation to the membrane. Therefore, the insulin receptor pathway may significantly affect cardiac glucose consumption. We assessed the expression of key metabolic enzymes in this pathway. Interestingly, Western-blot analysis showed meaningful upregulation of the PI3K protein (26%, *P* < 0.05, *n* = 7–12; Fig. 3) in PP2A-TG. AKT protein phosphorylation on Ser473 was increased non-significantly (95%, *P* = 0.05, *n* = 7–12; Fig. 3) compared to WT. PCR analysis showed substantial downregulation of Mtor (−22%, *P* < 0.05) in PP2A-TG and non-significantly decreased expression of AMPK encoded by the Prkaa2 gene (−20%, *P* = 0.09, *n* = 6–16) in PP2A-TG in comparison to WT (Table 2). Gene expression of Irs1, PI3K (Pik3ca gene) and Akt2 was not affected (Table 2). Expression of insulin receptor (Insr) was not affected on either the gene (Table 2) or the protein level (Fig. 3).

**Table 2:** Relative gene expressions of the genes of interest in the heart of the experimental animals

Gene	WT	PP2A-TG
Akt2	1.00 ± 0.3	0.78 ± 0.2
Gsk3b	1.00 ± 0.1	1.02 ± 0.2
Gys1	1.00 ± 0.1	0.74 ± 0.1*
Hk1	1.00 ± 0.1	1.15 ± 0.3
Hk2	1.00 ± 0.1	1.05 ± 0.2
Insr	1.00 ± 0.3	0.78 ± 0.2
Irs1	1.00 ± 0.4	0.89 ± 0.3
Mtor	1.00 ± 0.2	0.78 ± 0.2*
Pik3ca	1.00 ± 0.3	0.82 ± 0.2
Pparg	1.00 ± 0.3	0.59 ± 0.2*
Ppargca1	1.00 ± 0.2	0.72 ± 0.2*
Prkaa2	1.00 ± 0.2	0.80 ± 0.2^a^
Pygb	1.00 ± 0.3	0.86 ± 0.2
Pygm	1.00 ± 0.1	0.83 ± 0.2*
Slc2a1	1.00 ± 0.1	1.05 ± 0.3
Slc2a4	1.00 ± 0.2	0.82 ± 0.2^a^

Data are presented as mean ± standard deviation in percent of average CON expression levels (*n* = 6–16 per group, *p < 0.05 vs. WT;^a^0.1 > *P* > 0.05 vs. WT; For groups labelling, see Fig. 1

### PP2A did not significantly affect expression of glucose transporters

Reduced glucose consumption could be the result of altered expression of glucose transporters, which facilitate glucose transport. There are two glucose transporter proteins expressed in the heart: insulin-dependent GLUT4 and insulin-independent GLUT1. PCR analysis showed a non-significant decrease in the expression of the gene Slc2a4 encoding GLUT4 (−18%, *P* = 0.08, *n* = 6–16) and no alterations in the mRNA level of the gene Slc2a1 encoding GLUT1 glucose transporter between PP2A-TG and WT (Table 2). Protein expression showed no changes in either GLUT1 or GLUT4 expression. (Fig. 3).

### Expression of cardiac hexokinases and PFKFB2 does not influence reduced glucose consumption in PP2A-TG

**Fig. 4:**
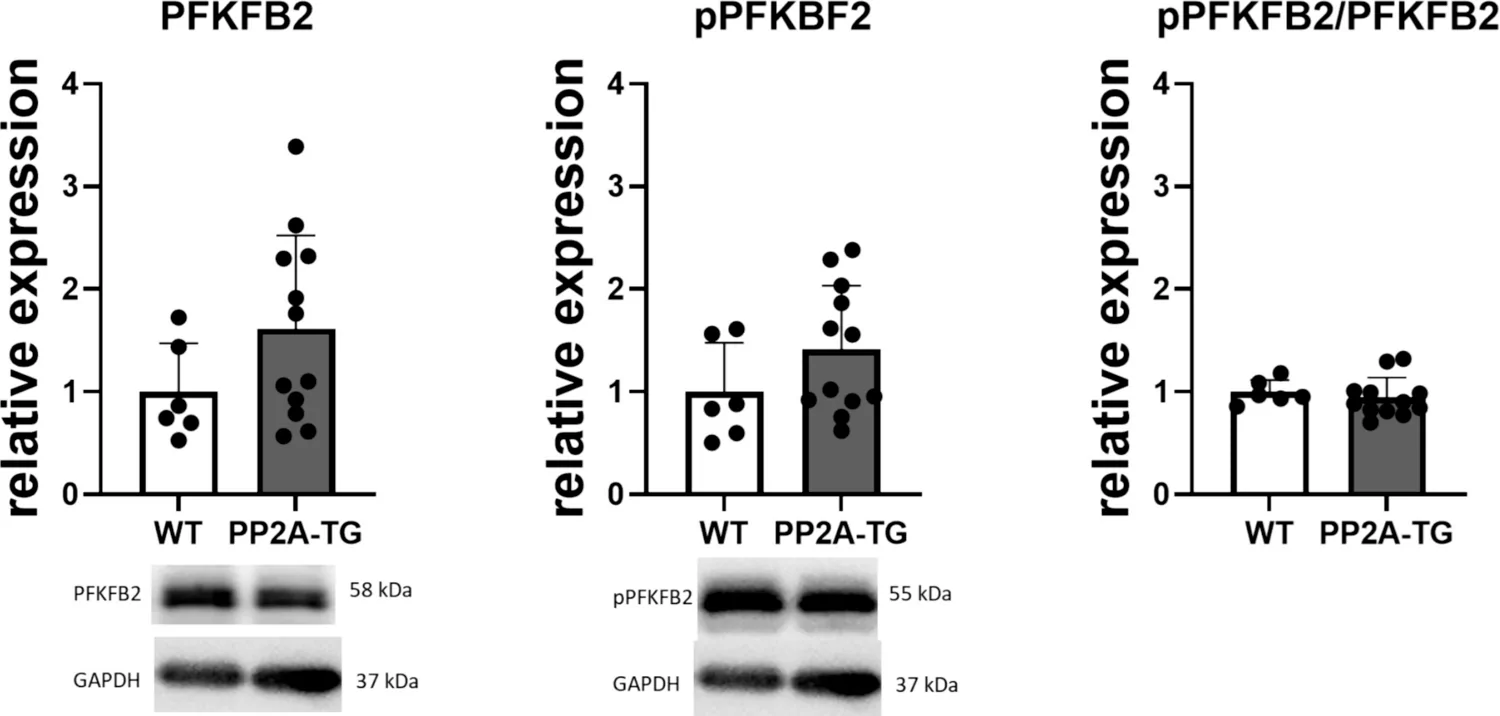
Western blot analysis of phosphorylated and non-phosphorylated PFKFB2 protein in the heart of WT and PP2A-TG mice with representative immunoblots. Data are presented as mean ± SD; *n* = 6–12

The first step of glycolysis is catalysed by the rate-limiting enzyme hexokinase. Thus, hexokinases control the initiation of all major pathways of glucose utilisation. These enzymes have an affinity for [^18^F]FDG, as well. Therefore, we measured the expression of hexokinase 1 and 2 to explain the changes in glucose consumption. Interestingly, we did not notice any differences between PP2A-TG and WT (Table 2). PFKFB2 is one of the key regulating enzymes of glycolysis catalysing the formation or degradation of fructose-2,6-bisphosphate. Its activity may be modulated by PP2A. We measured the protein expression of PFKFB2 and its phosphorylation at Ser483, which is associated with its activation. However, we did not detect any changes between the PP2A-TG and WT mice (Fig. 4).

### Downregulation of the key enzymes in glycogen metabolism without any alteration in glycogen content in the heart of PP2A-TG mice

**Fig. 5:**
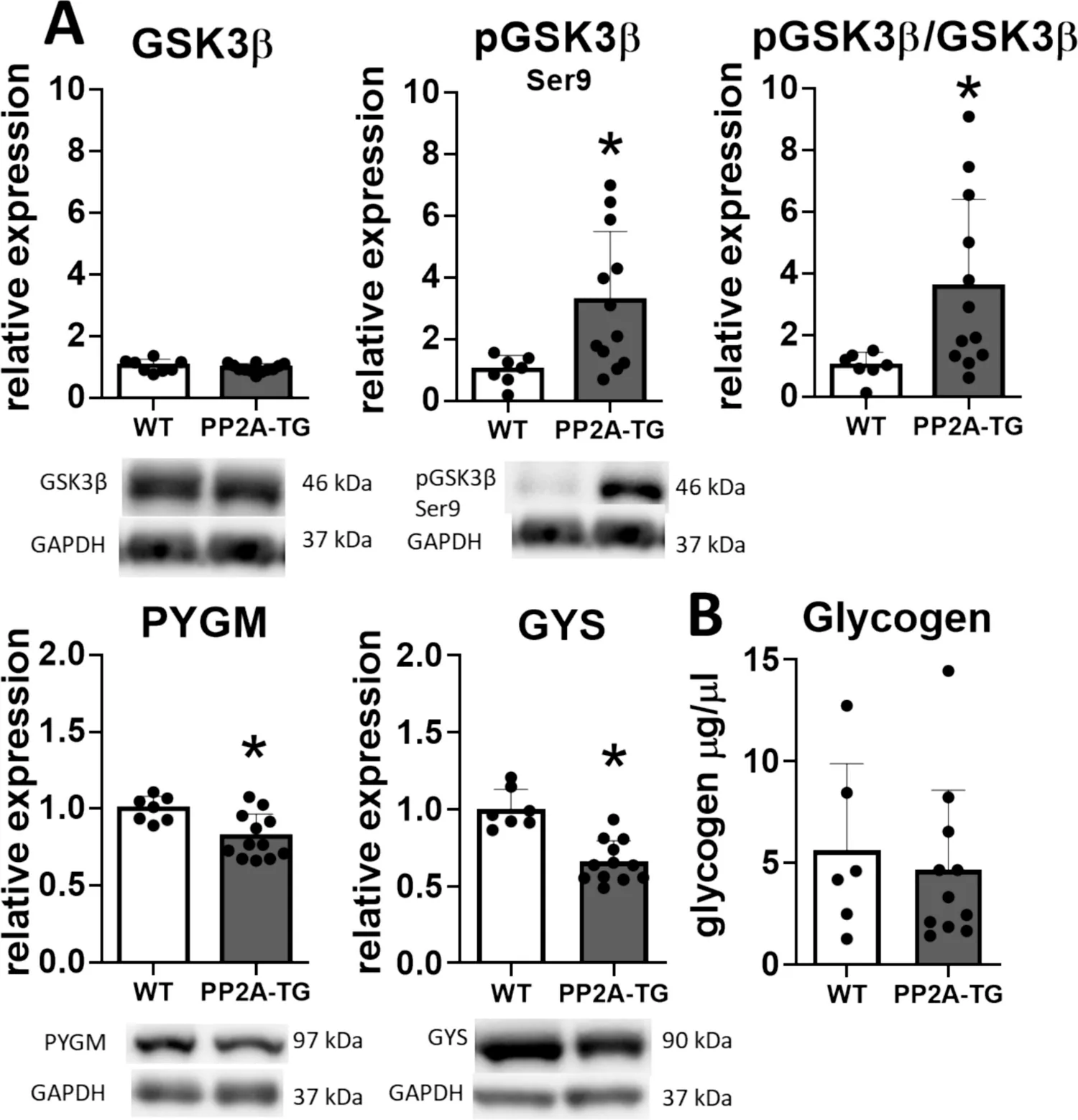
**A** Western blot analysis of glycogen metabolism-related proteins in the heart of WT and PP2A-TG mice with representative immunoblots. GSKβ phosphorylation was calculated as the pGSKβ and GSKβ expression ratio. Full-length blots are presented in Supplementary Figs. 3, 4 and 7 (**B**) Glycogen content in the heart of WT and PP2A-TG mice. Data are presented as mean ± SD; *n* = 7–12 per group; *P < 0.05 vs CON. For groups labelling, see Fig. 1

**Fig. 6:**
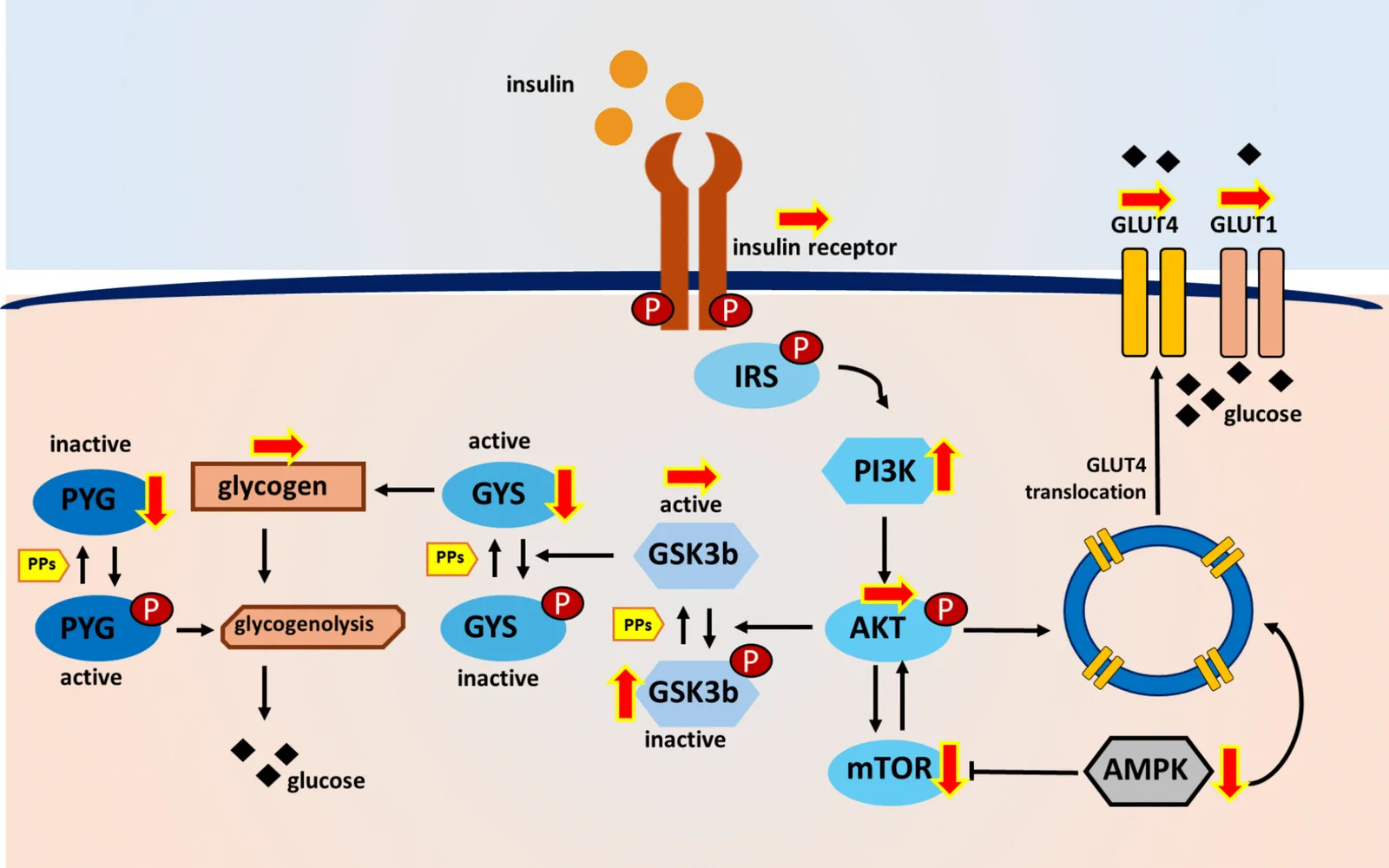
The scheme of intracellular insulin receptor pathway (Arneth et al. 2019; Umbarkar et al. 2023). Red arrows indicate the direction of the observed expression changes in proteins involved in glucose metabolism and regulation. AKT: Akt serine/threonine kinase; AMPK: AMP-activated; GLUT1, 4: glucose transporter 1, 4; Gsk3b: glycogen synthase kinase 3 beta; GYS: glycogen synthase; IRS: insulin receptor substrate; mTOR: mechanistic target of rapamycin kinase; PI3K: phosphatidylinositol-4,5-bisphosphate 3-kinase; PP: protein phosphatases; PYG: glycogen phosphorylase

Since glycogen serves as glucose storage for the heart, we studied phosphorylase as a key enzyme for glycogen conversion to glucose. Two isoforms, Pygm and Pygb, are present in the cardiac tissue. PCR analysis showed a decrease in Pygm gene expression (−17%; *P* < 0.05, *n* = 6–16) but not in Pygb in PP2A-TG mice (Table 2). Protein analysis of Pygm showed meaningful downregulation in PP2A-TG mice (−18%, *P* < 0.05, *n* = 7–12, Fig. 5A). On the other hand, glycogen synthase is the principal enzyme for glycogen synthesis (Fig. 6). We report here downregulation of the mRNA (−26%, *P* < 0.05, *n* = 6–16) as well as protein (−66%, *P* < 0.05, *n* = 7–12) of glycogen synthase in PP2A-TG compared to WT (Fig. 5A). Glycogen synthase kinase 3ß (GSK-3β) is the regulatory enzyme catalysing phosphorylation and thus inactivation of glycogen synthase (Fig. 6). The protein (Fig. 5A) and gene expression (Table 2) of GSK-3β did not change between groups. GSK-3β activity is inhibited by phosphorylation at Ser9. We report increased phosphorylation of GSK-3β at Ser9 (255%; *P* < 0.05, *n* = 7–12) indicating inactivation of the enzyme. Nevertheless, the analysis of total glycogen content in the heart samples did not show the changes between PP2A-TG and WT (Fig. 5B).

### Transcription factor PPARγ and its co-activator downregulation in PP2A-TG mice

Interestingly, the transcription factor PPARγ and its co-activator PGC-1α, encoded by the Ppargc1a gene, which are involved in regulation of the expression of proteins for glucose handling, was downregulated (41% and 28%, respectively; both *P* < 0.05, *n* = 6–16) in PP2A-TG compared to WT mice (Table 2).

## Discussion

Previously, we found structural and functional changes in the murine heart by detection of cardiac-specific overexpression of PP2Ac (Gergs et al. 2004). These changes can reasonably lead to altered cardiac metabolism (Minamimoto 2021). Here, we report a reduced myocardial glucose consumption displayed by [^18^F]FDG PET in PP2A-TG compared to WT mice, pointing out the significant role of PP2A in glucose metabolism of the heart. Alterations in [^18^F]FDG levels are known to occur in the diseased heart (Minamimoto 2021). In our previous experiments we found developed cardiac hypertrophy with isles of necrosis and fibrosis in PP2A-TG mice (Gergs et al. 2004). Other studies demonstrated reduced [^18^F]FDG uptake in cardiac hypertrophy (Handa et al. 2007), necrosis (Funabashi et al. 2006) and fibrosis (Marques-Alves et al. 2025). Moreover, in patients with congestive heart failure, the uptake rate of [^18^F]FDG was lower than in healthy controls (Taylor et al. 2001), indicating that increased expression of PP2A might be detrimental in patients with heart failure because it would impair energy production. Overall, PP2A gradually impairs cardiac function, leading to compensatory hypertrophy and resulting in an impairment of glucose uptake into the heart.

All functional PP2A holoenzymes are composed of a catalytic (C) subunit and a scaffolding subunit (A) emphasising their key role in enzymatic activity. This PP2A core dimer can further associate with a diverse array of regulatory B subunits, which determine the substrate specificity as well as subcellular localisation of the holoenzyme complex. Accordingly, when investigating the role of PP2A, targeting the catalytic and scaffolding subunits represents a rational strategy, as modulation at this level affects multiple holoenzyme assemblies, in contrast to transgenic approaches focused on individual B subunits (Reynhout and Janssens 2019). In mammals, two isoforms of the catalytic subunit (PP2Ac) have been identified: α and β. They are 97% identical and both are abundantly expressed in the heart. However, the α isoform (encoded by PPP2AC gene) is approximately ten-fold more abundant in mammalian heart compared to the β isoform (Gergs et al. 2004). Consequently, overexpression of PP2Ac is likely to exert a global effect on cardiac function, including metabolism, independently of the type of regulatory subunit.

We further investigated the underlying mechanisms behind the reduction in cardiac glucose consumption. Through its catalytic subunit, PP2A might directly dephosphorylate and inactivate enzymes involved in glucose uptake or glucose handling. Cardiac glucose uptake is regulated mainly by insulin (Nguyen et al. 1990), which binds to the insulin receptor and activates downstream receptor pathways that promote glucose uptake and utilisation. PP2A is involved in the negative regulation of this pathway, with a resulting effect on glucose homeostasis and insulin sensitivity. This regulatory relationship has been demonstrated in both liver and 3T3-L1 adipocytes (Ugi et al. 2004; Xiang et al. 2025). In our PP2A-TG model, insulin signalling in the heart is also affected, as indicated by upregulation of PI3K protein, downregulation of the Mtor gene, and a non-significant reduction in AMPK encoded by the Prkaa2 gene. Among the several components of this network PI3K/AKT are critical enzymes that regulate most actions of insulin (Xian et al. 2015). In liver, knocking out Ppp2cα results in enhanced phosphorylation of AKT, which leads to activation of insulin signalling. One would expect a decrease in phosphorylation of AKT in PP2A-TG mice but, intriguingly, in the heart of PP2A-TG mice, phosphorylation of the AKT protein is enhanced on Thr308 (Hoehn et al. 2015), and here we report a non-significant increase in phosphorylation on Ser473. Thus, increased phosphorylation of AKT could be an indirect effect of PP2A in this model, and we conclude that insulin receptor pathway effects are rather indirect in this model. However, they may contribute to attenuated glucose consumption in PP2A-TG heart.

Activation of insulin signalling results in GLUT4 translocation, followed by increased glucose uptake into the heart. Myocardial glucose uptake correlates with the gene expression of GLUT4 (Shoghi et al. 2008). Additionally, the insulin non-dependent glucose transporter GLUT1 is also expressed in the heart (Hadova et al. 2020). Changes in the expression of both could affect myocardial [^18^F]FDG uptake. However, we did not detect any changes in the expression of either of the GLUT proteins, although the expression of the Slc2a4 gene encoding GLUT4 tends to decrease. In fact, insulin is known to regulate GLUT4 translocation to the membrane rather than expression (Varma et al. 2018); therefore, the expression levels of GLUT4 in PP2A-TG might not refer exactly to glucose uptake into the heart. Nevertheless, this may further demonstrate an impact of PP2A on the insulin signalling pathway.

Following cellular uptake, glucose may enter glycolysis to produce some energy for the heart. The initial and most essential step of glycolysis is catalysed by hexokinases (Ashraf and Goyal 2026). There is some evidence that PP2A can impact the activity of the hexokinases (Dieni and Storey 2011). We studied the expression of two hexokinases, Hk1 and Hk2, but we did not find any changes in the hearts between WT and PP2A-TG mice. One of the key regulating enzymes in glycolysis modulated by PP2A (Tang et al. 2020) is 6-phosphofructo-2-kinase/fructose-2,6-biphosphatase 2 (PFKFB2) (Yuan et al. 2025). PFKFB2 activity is stimulated by AKT-mediated phosphorylation at Ser483. In cardiomyocytes, the increased phosphorylation of PFKFB2 at Ser483 has been associated with enhanced glucose consumption (Novellasdemunt et al. 2013). In our experimental model, neither total PFKFB2 expression nor phosphorylation at Ser483 was significantly altered. This observation may reflect the concurrent action of two opposing regulatory mechanisms affecting PFKFB2 phosphorylation in our model: enhanced AKT activity, which promotes PFKFB2 phosphorylation, and increased PP2A activity, which facilitates its dephosphorylation.

After cellular uptake, glucose may also be stored as glycogen via the glycogenesis pathway. Cardiac glycogen serves as a pivotal reserve for myocardial energy, and it may contribute to glucose homeostasis during hypoxia or ischemia (Xiang et al. 2025). Several studies suggest that either synthesis or degradation of glycogen can be regulated, at least in part, by PP2A (Clotet et al. 1995; Galbo et al. 2013); however, PP1 is considered to have the central role (Adeva-Andany et al. 2016). In the present study, we observed a downregulation of both glycogen synthase and glycogen phosphorylase expression in the hearts of PP2A-TG mice, without a concomitant change in total cardiac glycogen content. On one hand, glycogen synthase is a rate-limiting enzyme and plays a central role in glycogenesis (Buschiazzo et al. 2004). On the other hand, glycogen phosphorylase is the principal enzyme taking part in the first step of glycogenolysis and is thus responsible for the catabolism of glycogen (Migocka-Patrzałek and Elias 2021). The activities of both enzymes are reciprocally regulated by phosphorylation: glycogen synthase is activated, whereas glycogen phosphorylase is inactivated, by dephosphorylation (Adeva-Andany et al. 2016), and PP2A could participate in this dephosphorylation. Downregulation of both enzymes in PP2A-TG animals point out the effect of PP2A on glycogen metabolism in the heart. We conclude that PP2A alters the expression of key enzymes in glycogen metabolism, whereas the expression of hexokinases is not affected. These changes may contribute to observed reduction in myocardial glucose uptake in PP2A-TG mice.

Glycogen synthase is a tightly regulated enzyme and is known to be inhibited by phosphorylation via glycogen synthase kinase 3 beta (GSK3β). Unexpectedly, we previously noted (Hoehn et al. 2015) and confirm here an increased phosphorylation of GSK3β at serine 9 in PP2A-TG hearts, which is associated with its inactivation. This finding is surprising, as PP2A has been proposed to dephosphorylate GSK3β at Ser9, thereby enhancing its activity (Lee et al. 2005; Chu et al. 2015). We attribute this effect to observed enhanced phosphorylation and thus augmented activity of the AKT kinase in PP2A-TG (Hoehn et al. 2015), which is known to further phosphorylate GSK3β. In this context, the activity of glycogen synthase should be increased and produce more glycogen. However, we did not detect increased glycogen content, suggesting the presence of additional mechanisms that maintain glycogen content stable in PP2A-TG heart. Such compensatory mechanisms may involve altered expression patterns of key enzymes involved in glycogen metabolism. This notion is supported by the observed downregulation of both glycogen synthase and glycogen phosphorylase in PP2A-TG mice. Although regulation via PP1 represents a plausible (Gergs et al. 2004) contributing factor, investigation of this pathway showed no changes in PP1 activity between WT and TG mice (Gergs et al. 2004). In addition, the roles of regulatory B subunits of PP2A could be considered; however, this was beyond the scope of the present study. Collectively, these alterations in glycogen metabolism may be associated with a reduction in glucose consumption in the hearts of PP2A-TG mice.

Peroxisome proliferator-activated receptor gamma (PPARγ) plays an integrated role in controlling the expression of genes affecting metabolism, morphogenesis and inflammatory response (Janani and Ranjitha Kumari 2015). In the adult heart, PPARγ is implicated in the regulation of basal myocardial fatty acid utilisation (Luo et al. 2010), and pharmacological activation of PPARγ improves insulin sensitivity (Janani and Ranjitha Kumari 2015). The observed downregulation of PPARγ in PP2A-TG mice, together with its coactivator PGC-1α downregulation, may result in the impaired cardiac structure and metabolism (Luo et al. 2010; Chen et al. 2021) and could be responsible for changes in glucose handling in the hearts of PP2A-TG mice, although the literature remains inconsistent on this point (Barbieri et al. 2012). The involvement of PP2A in the modulation of PPARγ and PGC-1α activity appears to be indirect (Altiok et al. 1997; Mohamed Khalil et al. 2019). Nonetheless, reduced expression of this transcriptional regulatory axis further supports our findings of disrupted metabolic regulation in PP2A-TG hearts.

The present study has several limitations. Due to the limited spatial resolution of the scan, the precise cellular localisation of [^18^F]FDG within the myocardium cannot be fully resolved. [^18^F]FDG could be transported into cardiomyocytes but also into the non-myocyte cell populations in the heart. As most of the cardiac mass is composed of cardiomyocytes (Zhou and Pu 2016), we assume that we have measured the glucose consumption in cardiomyocytes. Furthermore, we did not measure the phosphorylation status of all putatively involved proteins. Several proteins involved in glucose metabolism are candidates for these kinds of studies, which were, however, beyond the scope of the present work, which wanted to establish a proof of principle. Finally, the mice were not subject to fasting prior to euthanasia due to ethical considerations, as fasting for several hours was not approved by the institutional animal care and use committee. Starving might reasonably influence the glycaemic status of mice and further processes in glucose metabolism, but all mice had the same access to food; therefore, the metabolic status should be similar. In the present study, we primarily focused on cardiac glucose metabolism, as reduced glucose utilisation is closely associated with alterations in glucose handling and overall metabolic regulation. However, cardiac metabolism is governed by a highly interconnected network of pathways, whereby perturbations in a single physiological process may exert widespread effects on multiple metabolic routes. In this context, it is reasonable to hypothesise that the altered metabolic state of the heart observed in our model may also influence free fatty acid (FFA) metabolism, which represents the predominant energy source for the myocardium. Conversely, the observed reduction in glucose consumption may be secondary to PP2A-mediated modulation of FFA metabolism. Enhanced fatty acid oxidation is known to suppress glycolysis through an increase in cytosolic citrate levels, a mechanism classically described as the Randle Cycle (Stanley et al. 2005). Nevertheless, whether PP2A directly promotes FFA oxidation remains unclear. Existing evidence suggests that PP2A may influence lipid metabolism via dephosphorylation of AMP-activated protein kinase (AMPK), a key regulator of cellular energy homeostasis and lipid metabolic pathways (Joseph et al. 2015; Yang et al. 2016). Further investigations are therefore required to elucidate the role of PP2A in the regulation of additional metabolic pathways and to clarify its broader impact on cardiac energy metabolism.

## Conclusion

In this work, we report diminished glucose consumption by the heart of PP2A-TG mice, which was accompanied by changes in expression or phosphorylation of the proteins involved in insulin-dependent glucose uptake, namely PI3K/AKT/m-TOR, proteins participating in glycogen metabolism (GYS, PYGM and GSK3β) and decreased gene expression of PPARγ and its coactivator PGC-1α regulating the expression of genes involved in cardiac metabolism. We suggest that metabolic changes are due to impaired cardiac structure and function in PP2A-TG, and the effect of PP2A on cardiac glucose consumption is rather indirect but significant and may further influence the function of the heart. The model might be translated into humans since it is well in line with clinical results reporting lower [^18^F]FDG uptake rate in patients with congestive heart failure. Influencing glucose metabolism and handling could be useful in many cardiac pathologies, including heart failure and ischemic heart disease (Shao and Tian 2015). PP2A could be a novel potential target in cardiovascular diseases as there are no current drugs targeted against PP2A, although many studies report its significant role in the diseased heart.

## Supplementary Information

Below is the link to the electronic supplementary material.

ESM 1 (PDF 1.38 MB)

## Data Availability

All source data for this work (or generated in this study) are available upon reasonable request.
