# Supplementary material for: Protein phosphatase 2A impairs cardiac uptake of glucose in the mammalian heart
Source: Naunyn Schmiedebergs Arch Pharmacol. 2026 Jun 25;399(12):19489–501. doi: 10.1007/s00210-026-05617-x (PMC13593684; doi:10.1007/s00210-026-05617-x)
Supplement: Supplementary file 1 [file 210_2026_5617_MOESM1_ESM.pdf]

Supplementary material

Title: Protein phosphatase 2A impairs cardiac uptake of glucose in the mammalian heart

Journal: Naunyn-Schmiedeberg's Archives of Pharmacology

Katarina Hadova[1], Daniel Gündel[2], Torsten Knieß[3], Peter Brust[2,4] Jan Klimas[1], Joachim Neumann[5], Ulrich Gergs[5*]

***Author for Correspondence:** Ulrich Gergs, Institut für Pharmakologie und Toxikologie, Medizinische Fakultät, Martin-Luther-Universität Halle-Wittenberg, Magdeburger Str. 4, 06097 Halle, Germany
Tel.: +49-345-557 4093
Fax: +49-345-557 1835
E-mail: ulrich.gergs@medizin.uni-halle.de

Supp. 1a

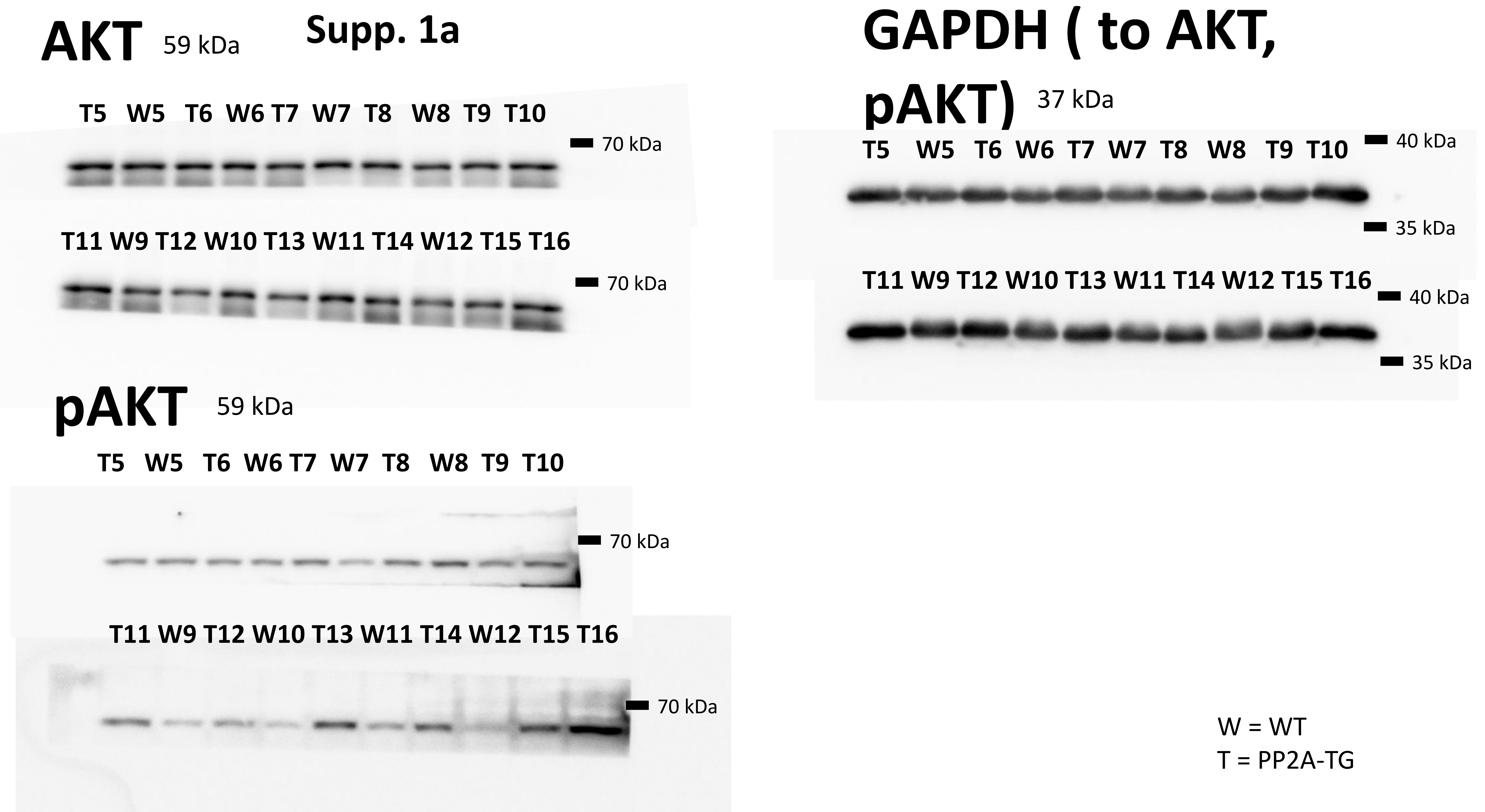

AKT 59 kDa
T5 W5 T6 W6 T7 W7 T8 W8 T9 T10
70 kDa
T11 W9 T12 W10 T13 W11 T14 W12 T15 T16
70 kDa
pAKT 59 kDa
T5 W5 T6 W6 T7 W7 T8 W8 T9 T10
70 kDa
T11 W9 T12 W10 T13 W11 T14 W12 T15 T16
70 kDa
GAPDH (to AKT, pAKT) 37 kDa
T5 W5 T6 W6 T7 W7 T8 W8 T9 T10
40 kDa
35 kDa
T11 W9 T12 W10 T13 W11 T14 W12 T15 T16
40 kDa
35 kDa
W = WT
T = PP2A-TG

AKT 59 kDa

Supp. 1b - marker

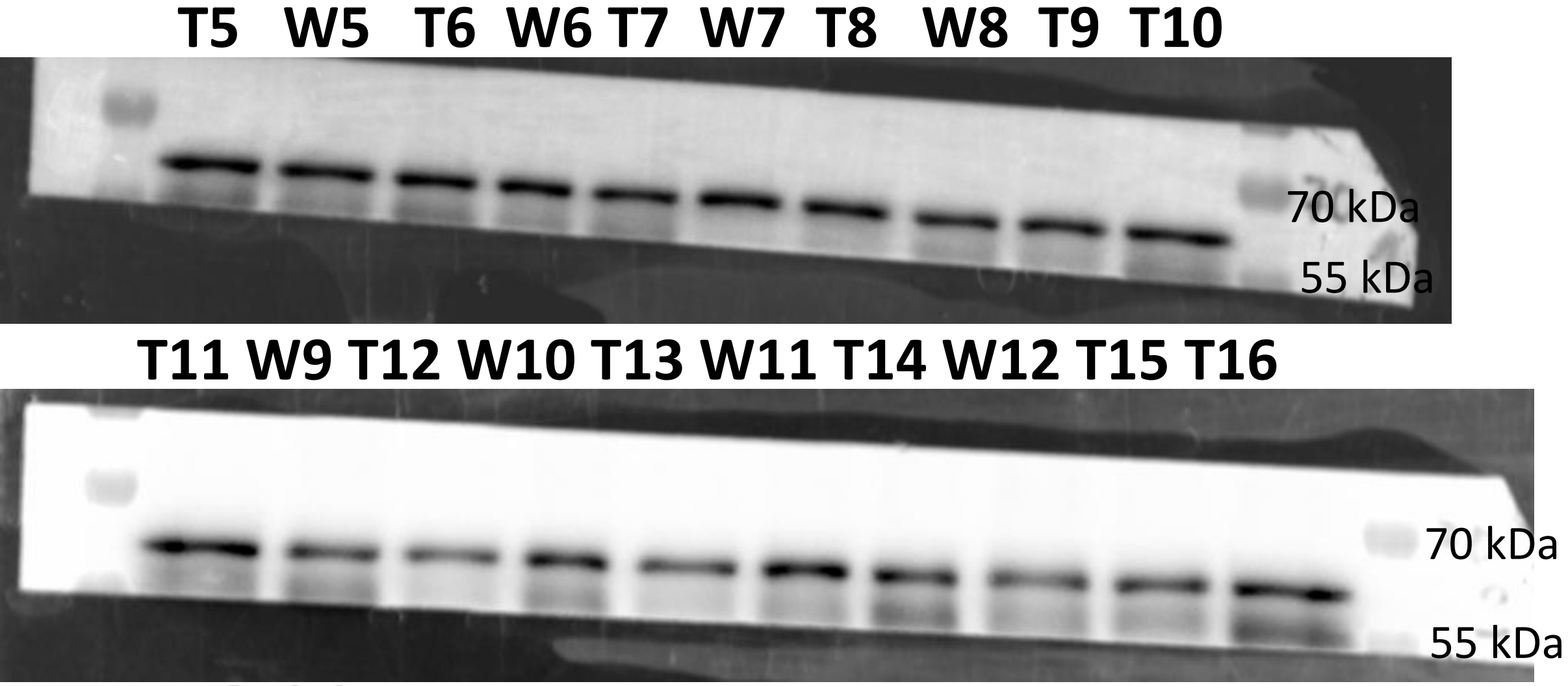

pAKT 59 kDa

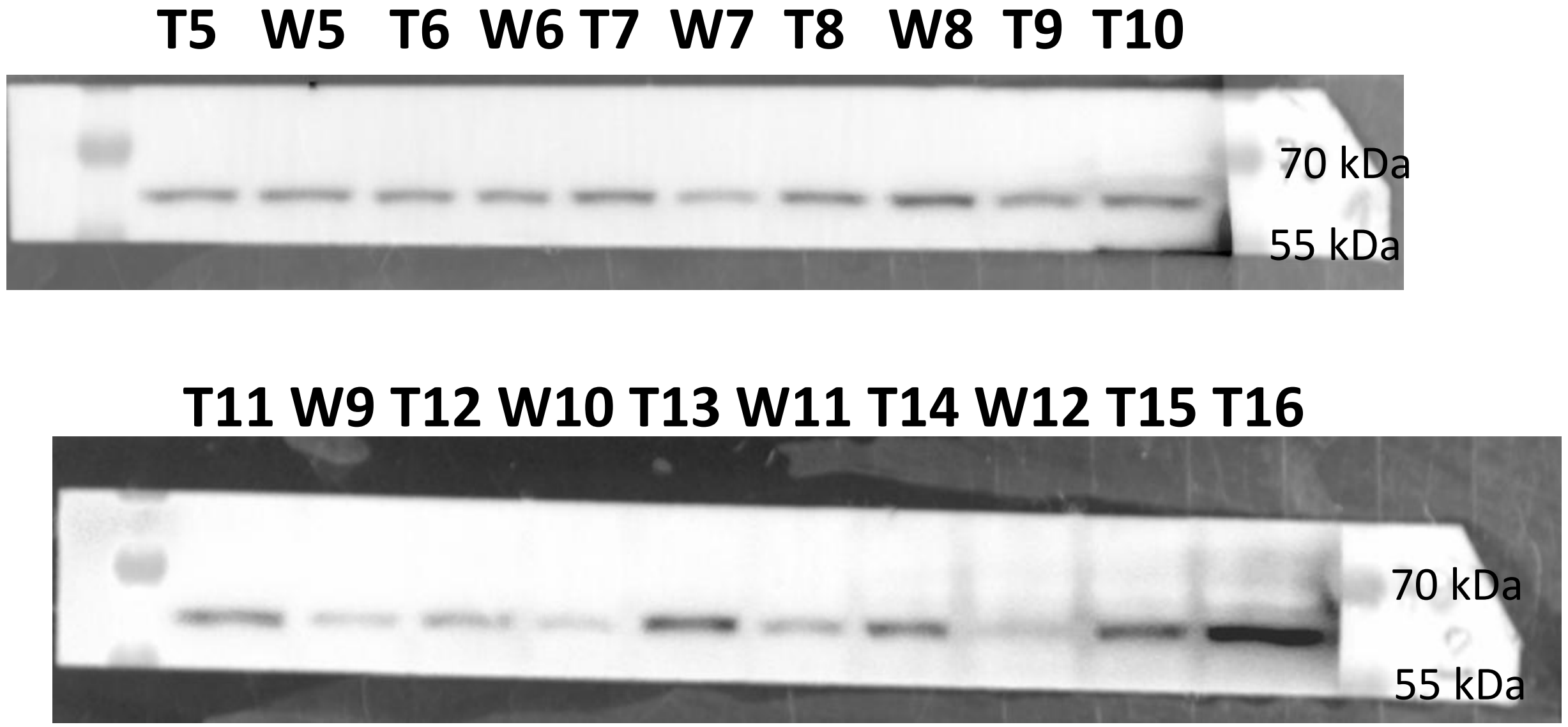

GAPDH (to AKT, pAKT)

37 kDa

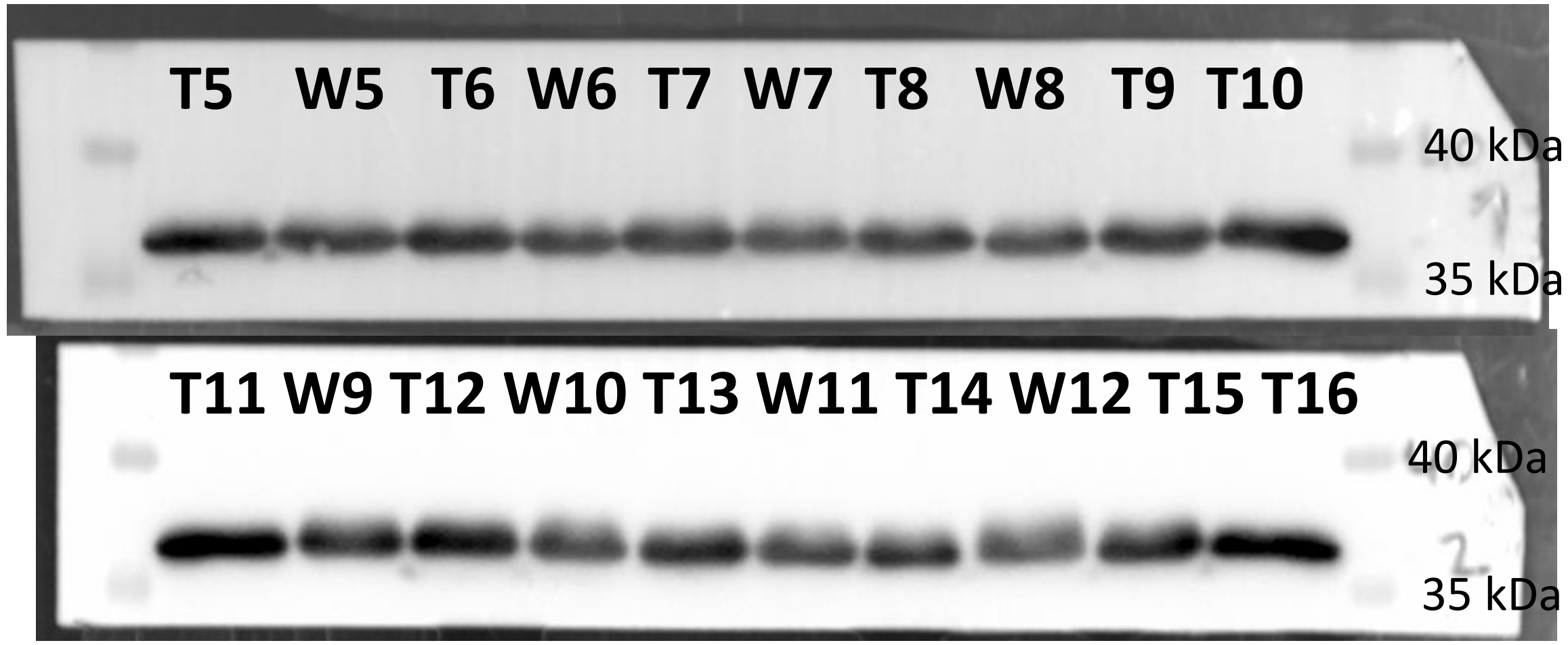

W = WT
T = PP2A-TG

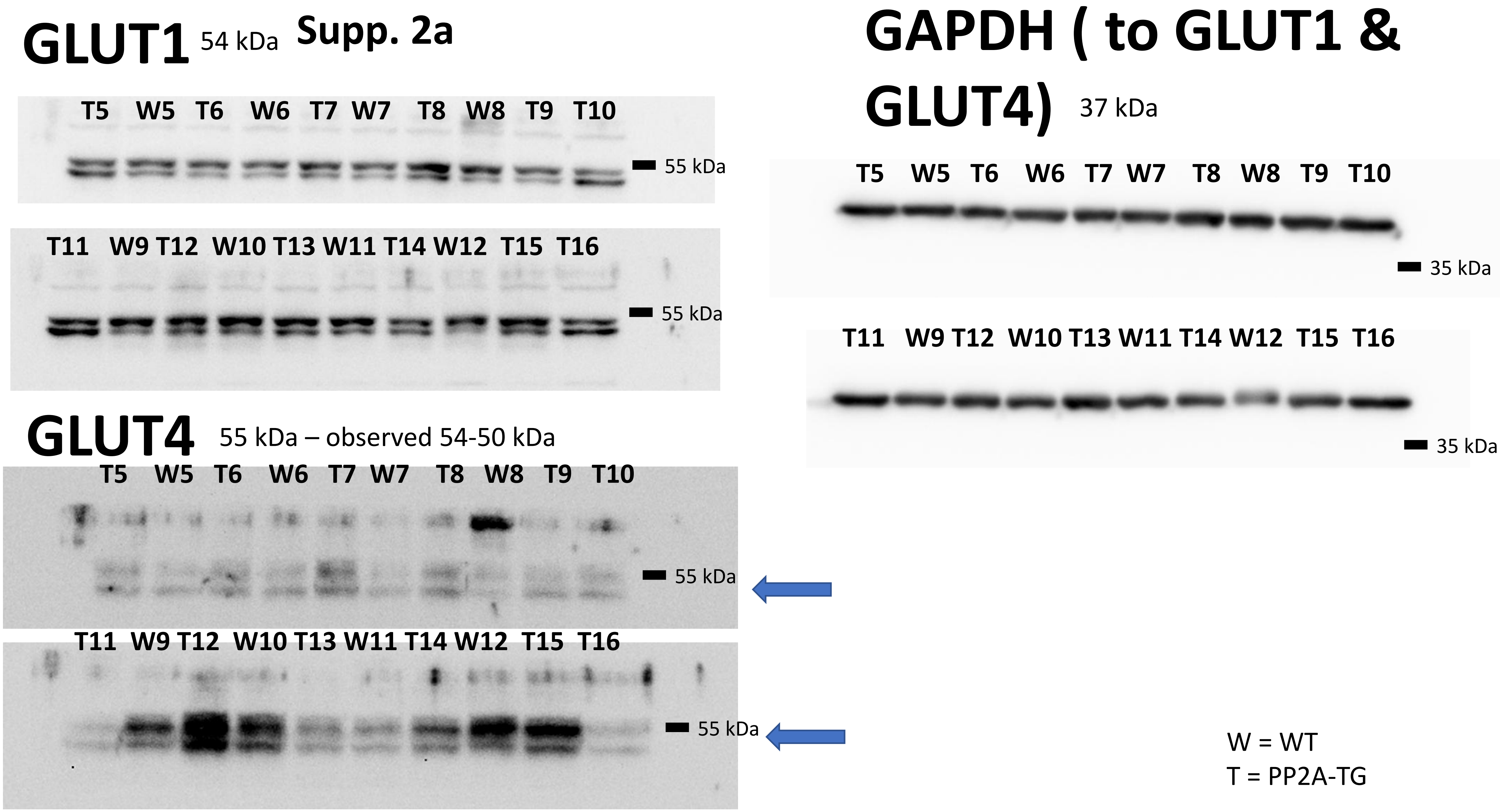

GLUT1 54 kDa
Supp. 2a
T5 W5 T6 W6 T7 W7 T8 W8 T9 T10
55 kDa
T11 W9 T12 W10 T13 W11 T14 W12 T15 T16
55 kDa
GLUT4 55 kDa – observed 54-50 kDa
T5 W5 T6 W6 T7 W7 T8 W8 T9 T10
55 kDa
T11 W9 T12 W10 T13 W11 T14 W12 T15 T16
55 kDa
GAPDH (to GLUT1 & GLUT4) 37 kDa
T5 W5 T6 W6 T7 W7 T8 W8 T9 T10
35 kDa
T11 W9 T12 W10 T13 W11 T14 W12 T15 T16
35 kDa
W = WT
T = PP2A-TG

GLUT1 54 kDa Supp. 2b - marker

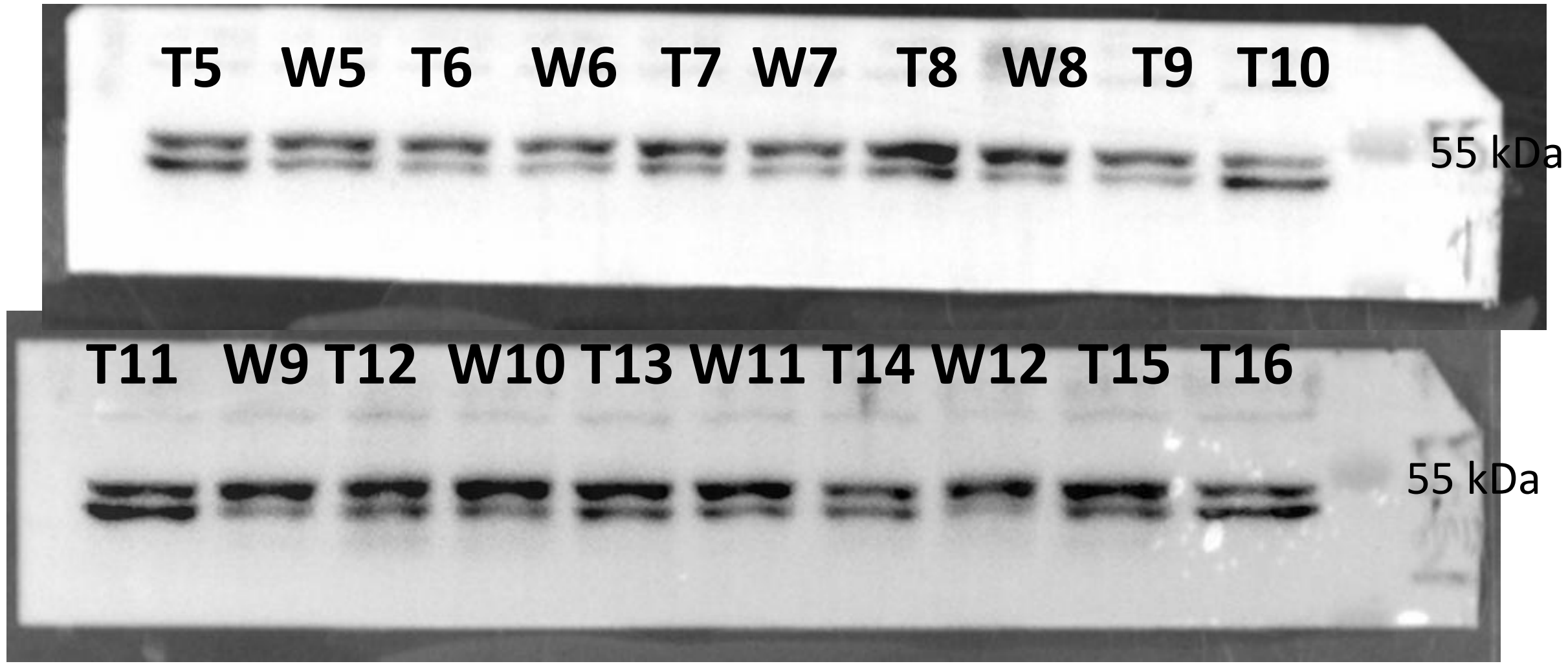

GLUT4 55 kDa – observed 54-50 kDa

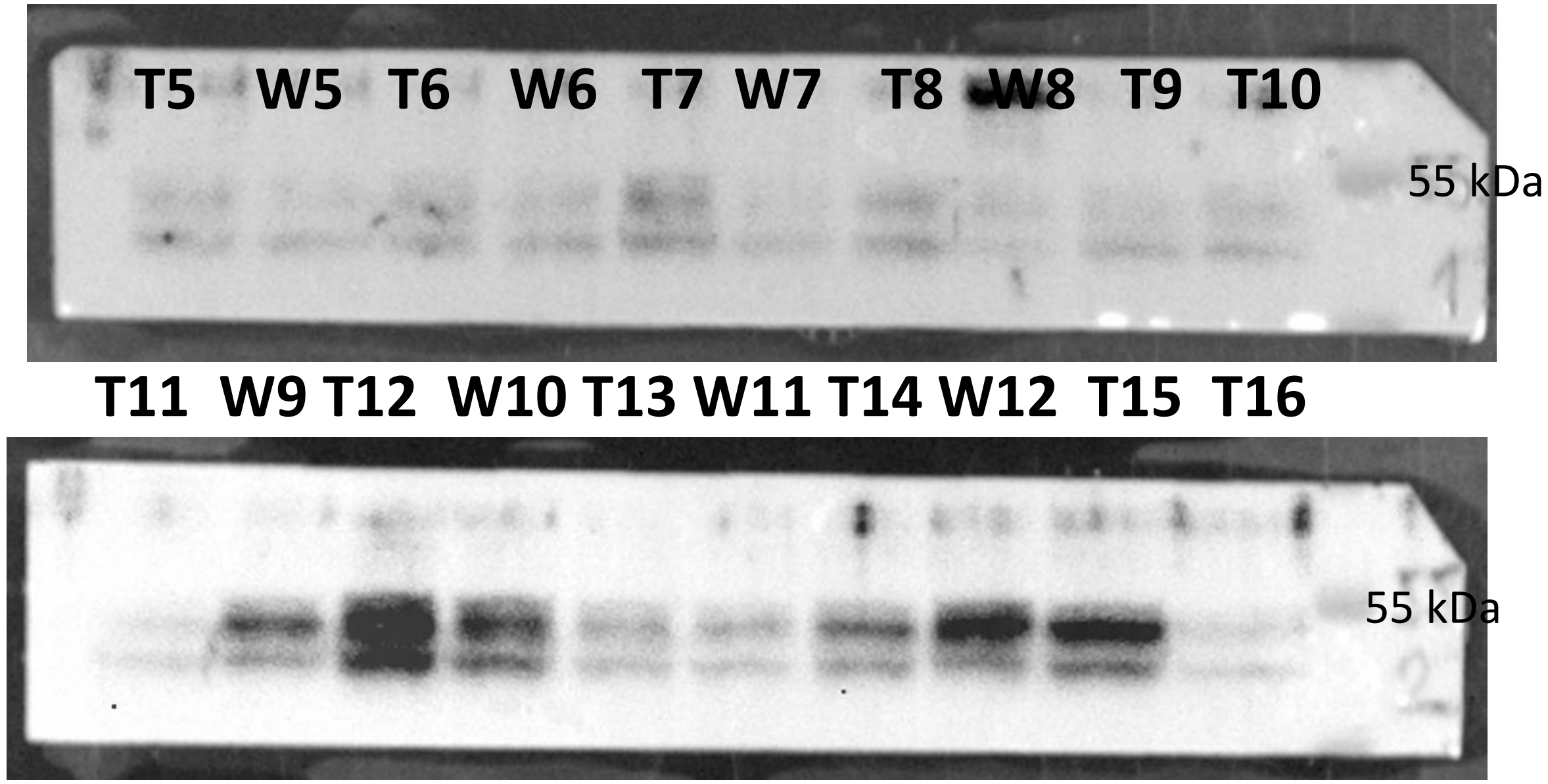

GAPDH (to GLUT1 & GLUT4) 37 kDa

T5 W5 T6 W6 T7 W7 T8 W8 T9 T10

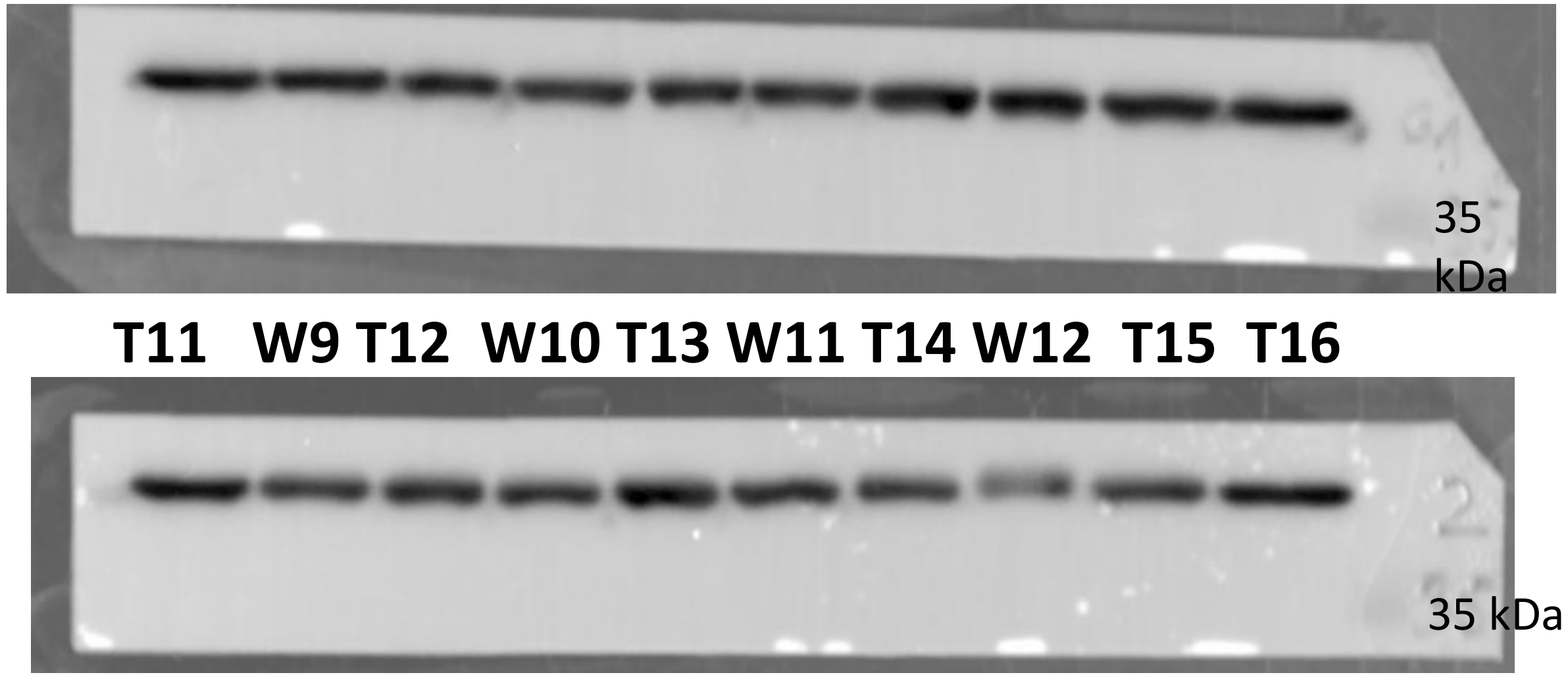

W = WT
T = PP2A-TG

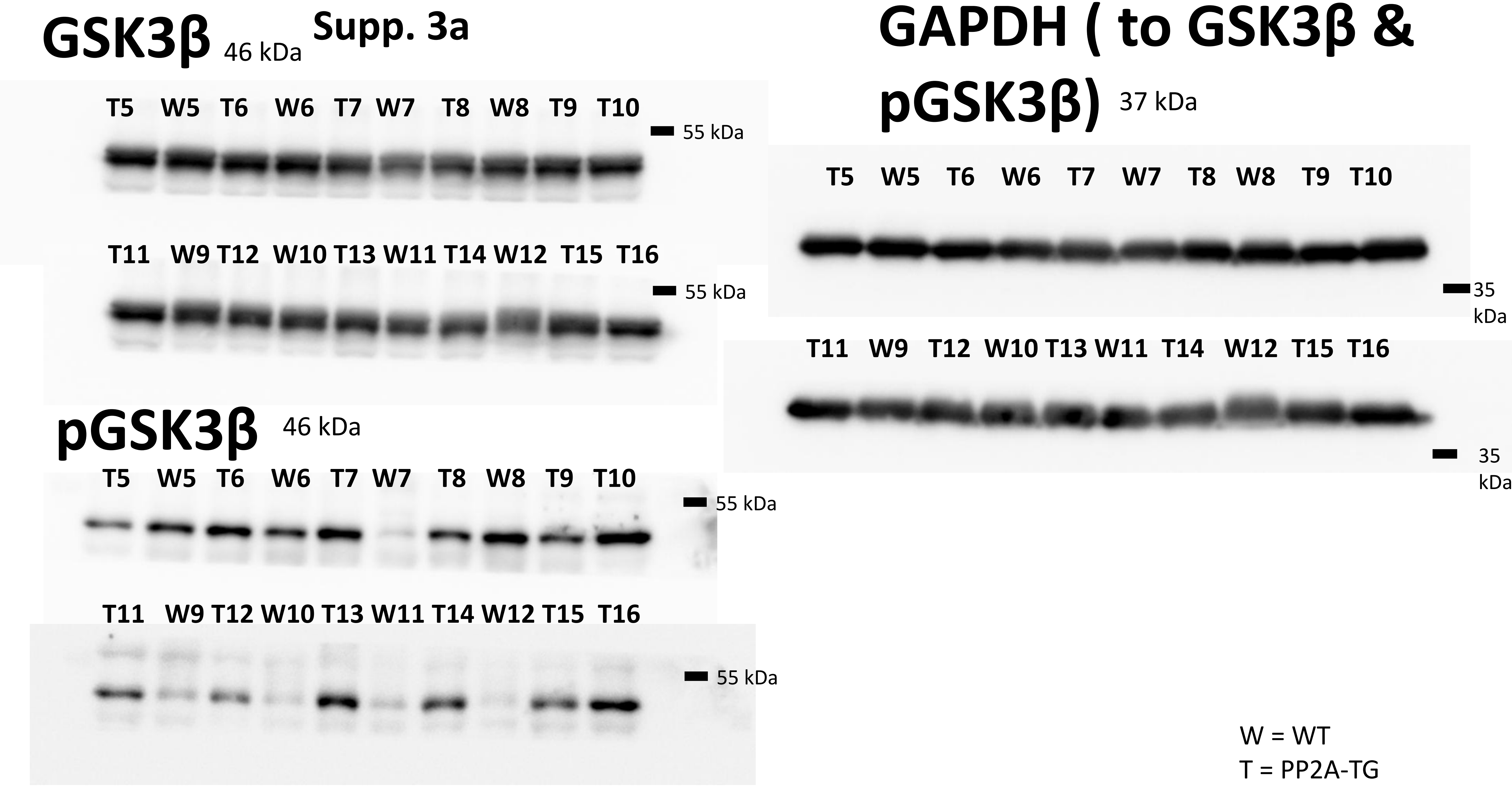
GSK3β 46 kDa
Supp. 3a
T5 W5 T6 W6 T7 W7 T8 W8 T9 T10
55 kDa
T11 W9 T12 W10 T13 W11 T14 W12 T15 T16
55 kDa
pGSK3β 46 kDa
T5 W5 T6 W6 T7 W7 T8 W8 T9 T10
55 kDa
T11 W9 T12 W10 T13 W11 T14 W12 T15 T16
55 kDa
GAPDH (to GSK3β & pGSK3β) 37 kDa
T5 W5 T6 W6 T7 W7 T8 W8 T9 T10
35 kDa
T11 W9 T12 W10 T13 W11 T14 W12 T15 T16
35 kDa
W = WT
T = PP2A-TG

GSK3β 46 kDa Supp. 3b -marker

T5 W5 T6 W6 T7 W7 T8 W8 T9 T10

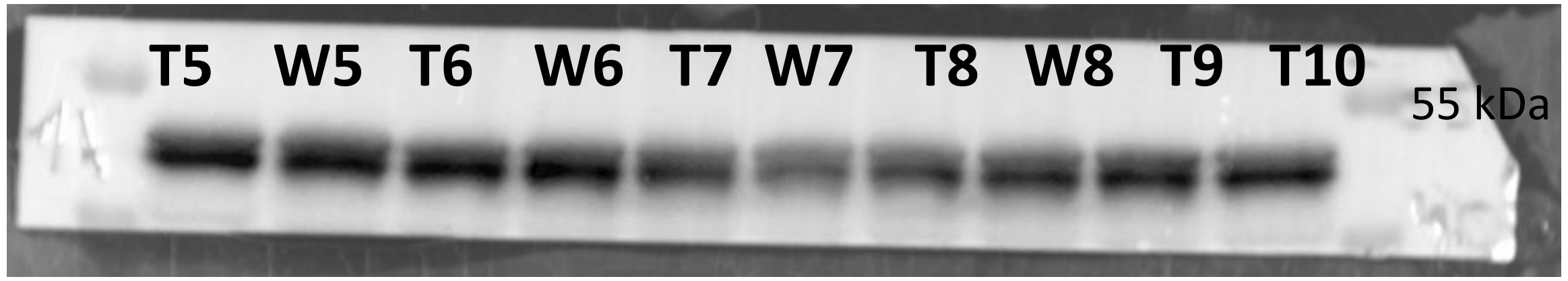

55 kDa

T11 W9 T12 W10 T13 W11 T14 W12 T15 T16

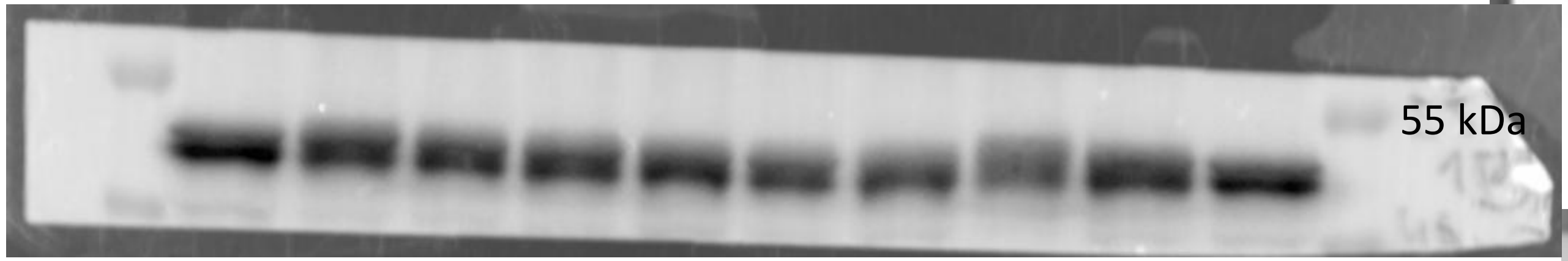

55 kDa

pGSK3β 46 kDa

T5 W5 T6 W6 T7 W7 T8 W8 T9 T10

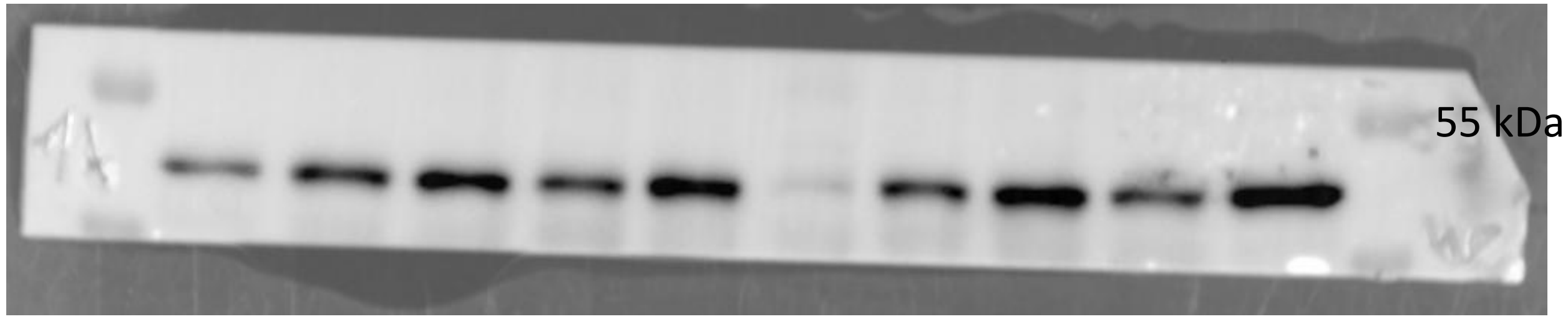

55 kDa

T11 W9 T12 W10 T13 W11 T14 W12 T15 T16

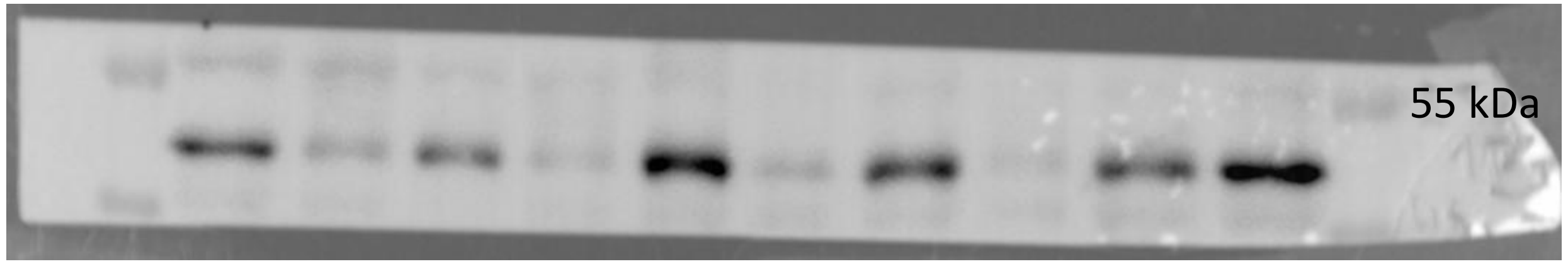

55 kDa

GAPDH (to GSK3β & pGSK3β) 37 kDa

T5 W5 T6 W6 T7 W7 T8 W8 T9 T10

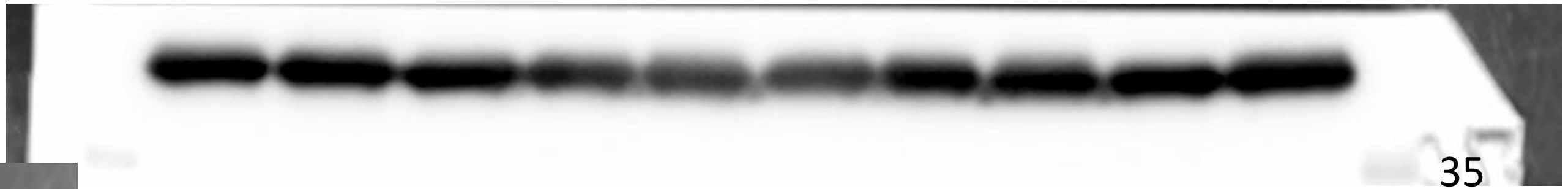

35 kDa

T11 W9 T12 W10 T13 W11 T14 W12 T15 T16

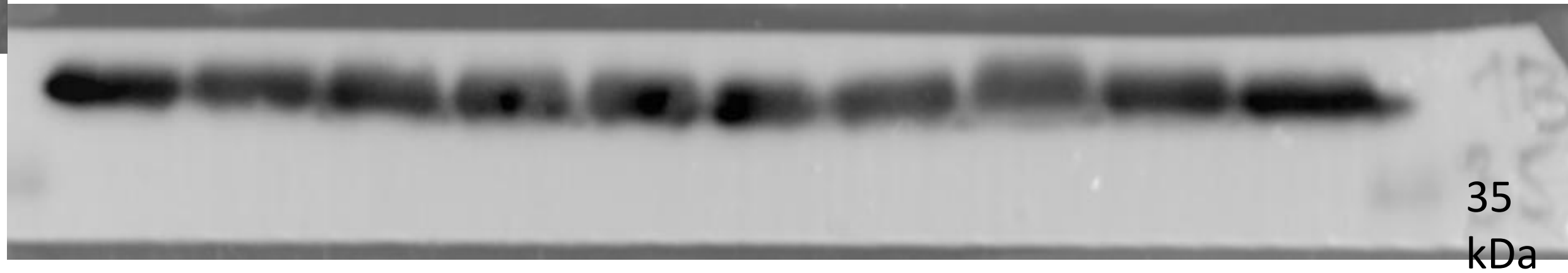

35 kDa

W = WT
T = PP2A-TG

GYS 90 kDa

Supp. 4a

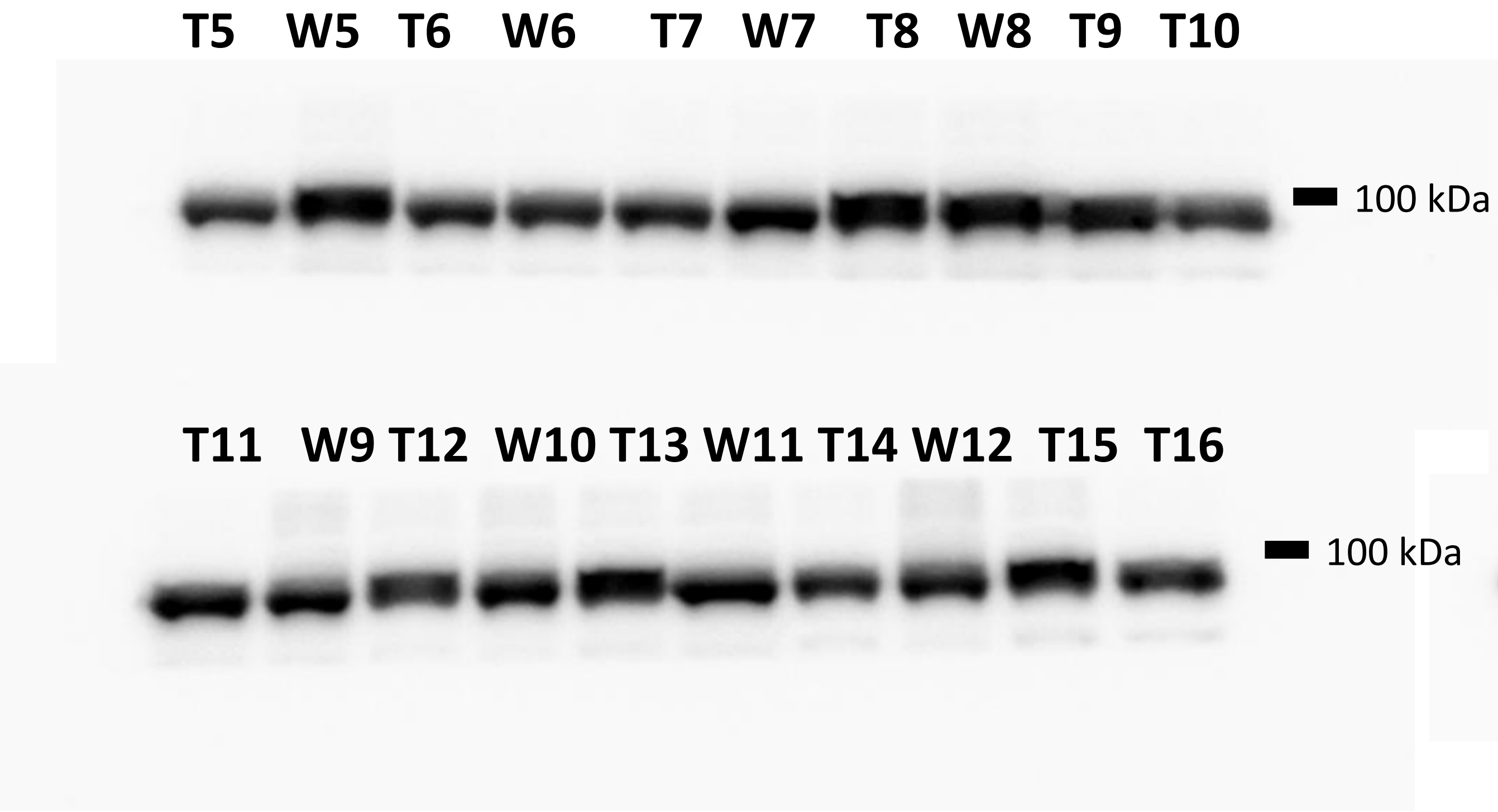

GAPDH (to GYS) 37 kDa

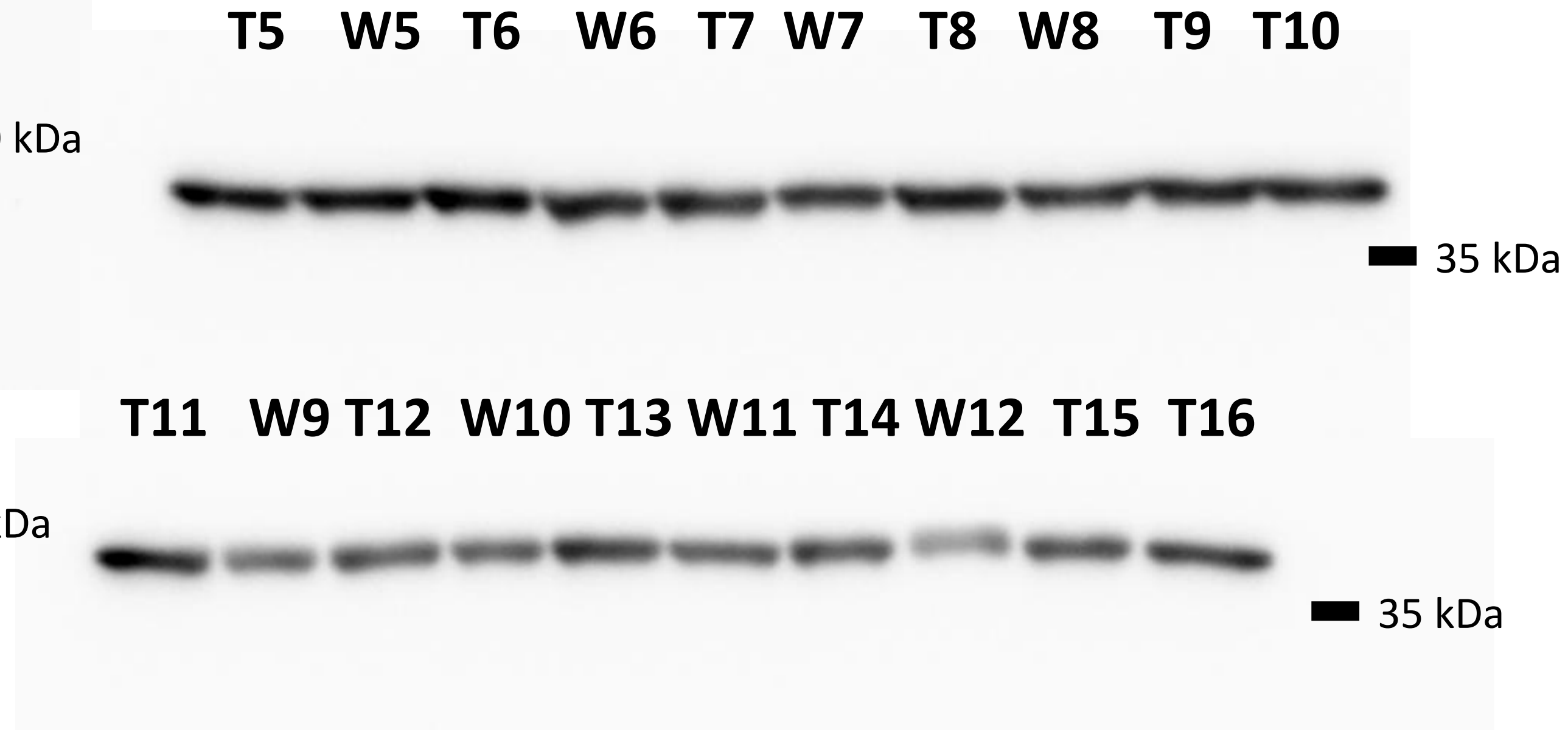

W = WT
T = PP2A-TG

GYS 90 kDa

Supp. 4b - marker

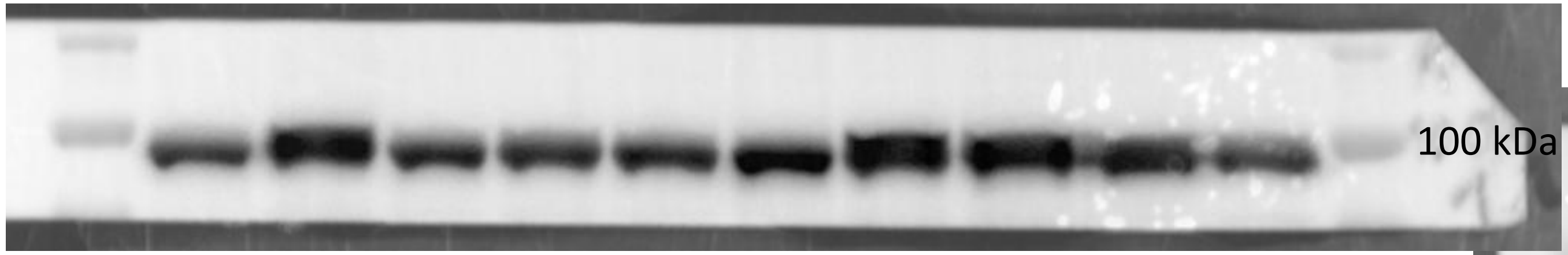

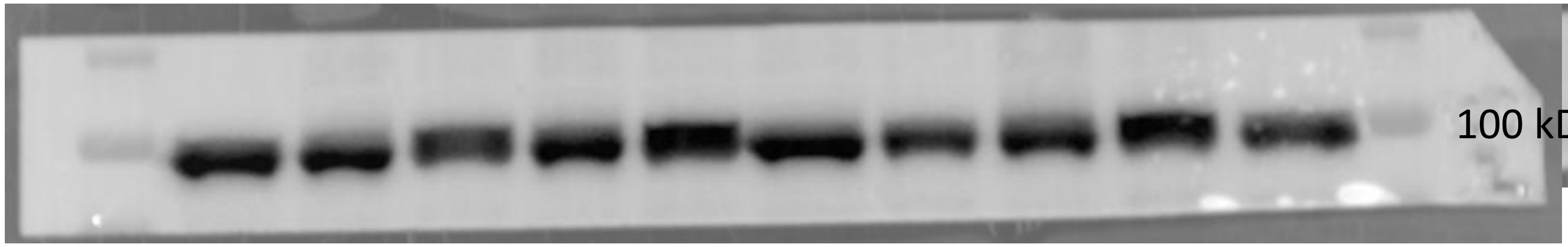

GAPDH (to GYS) 37 kDa

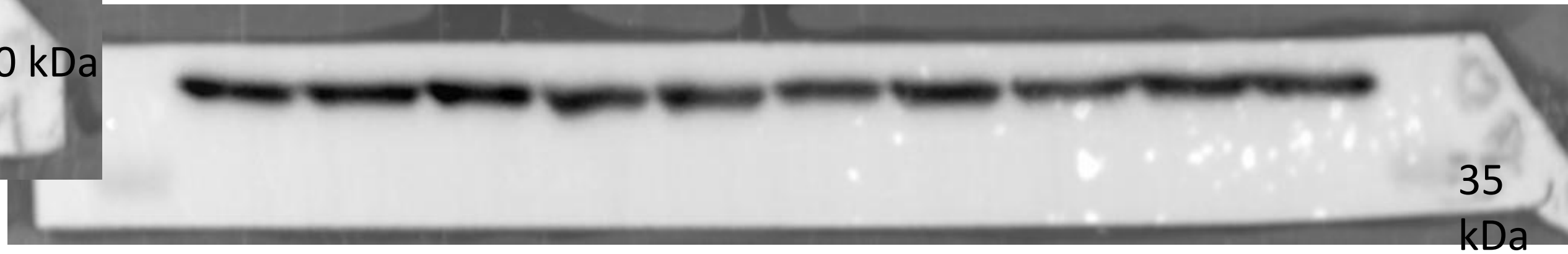

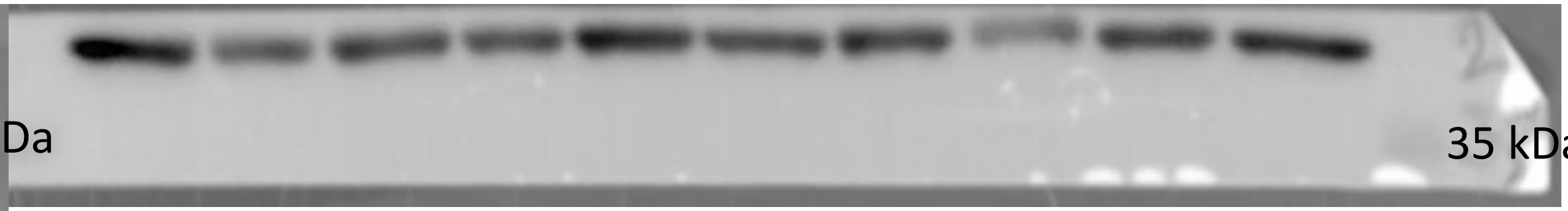

W = WT

T = PP2A-TG

INSR 95 kDa **Supp. 5a**

GAPDH (to INSR) 37 kDa

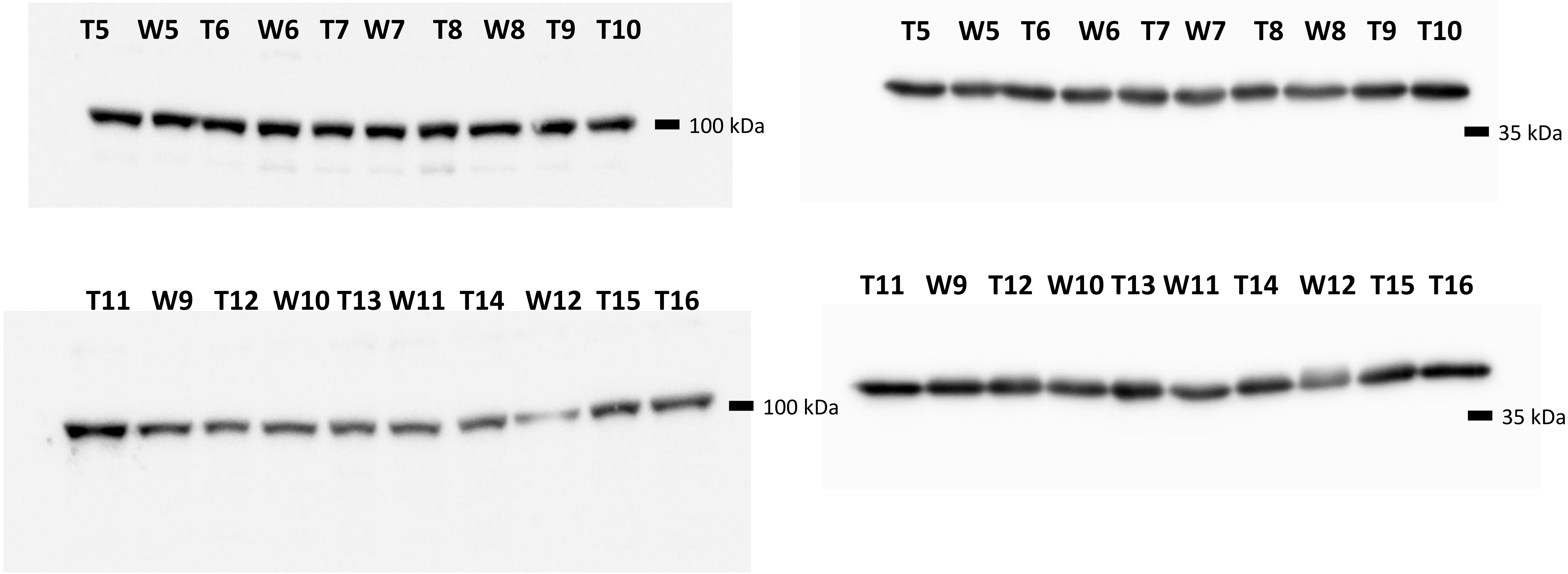

W = WT
T = PP2A-TG

Supp. 5b - marker

INSR

95 kDa

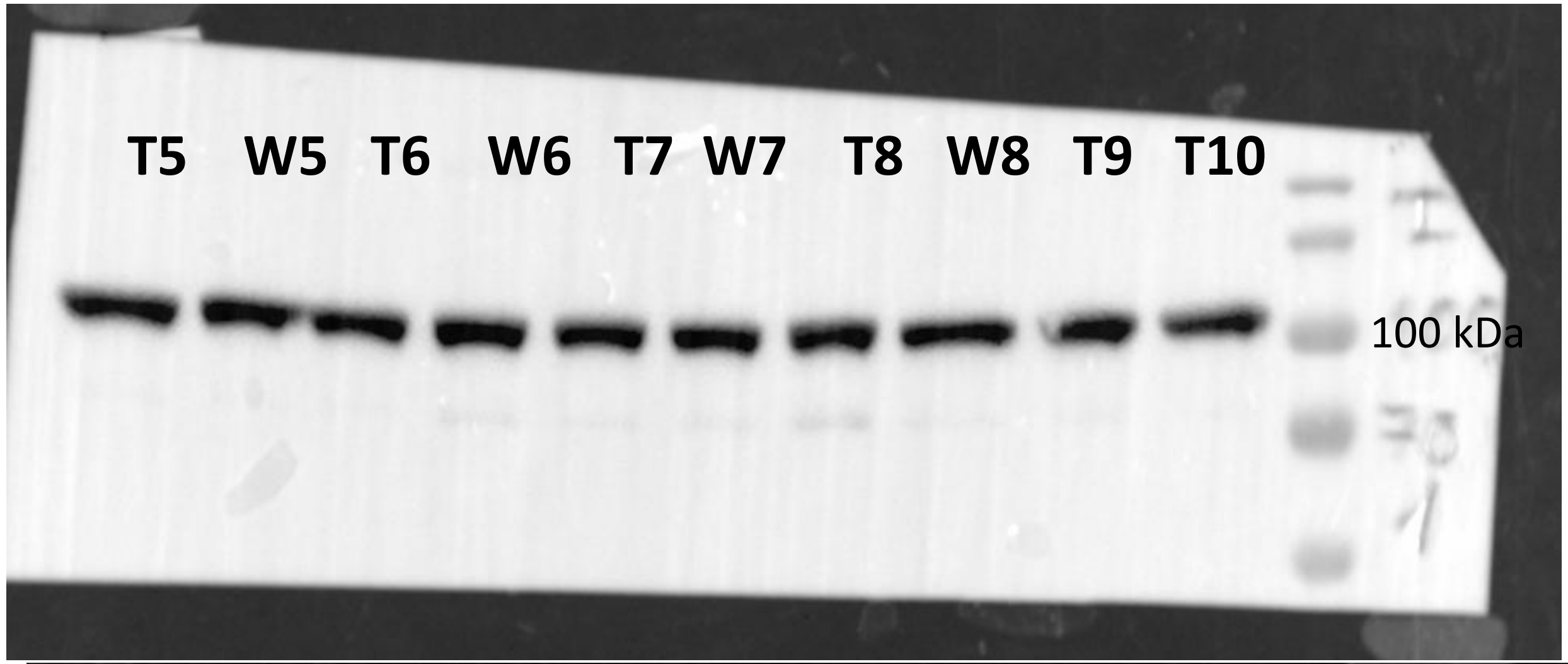

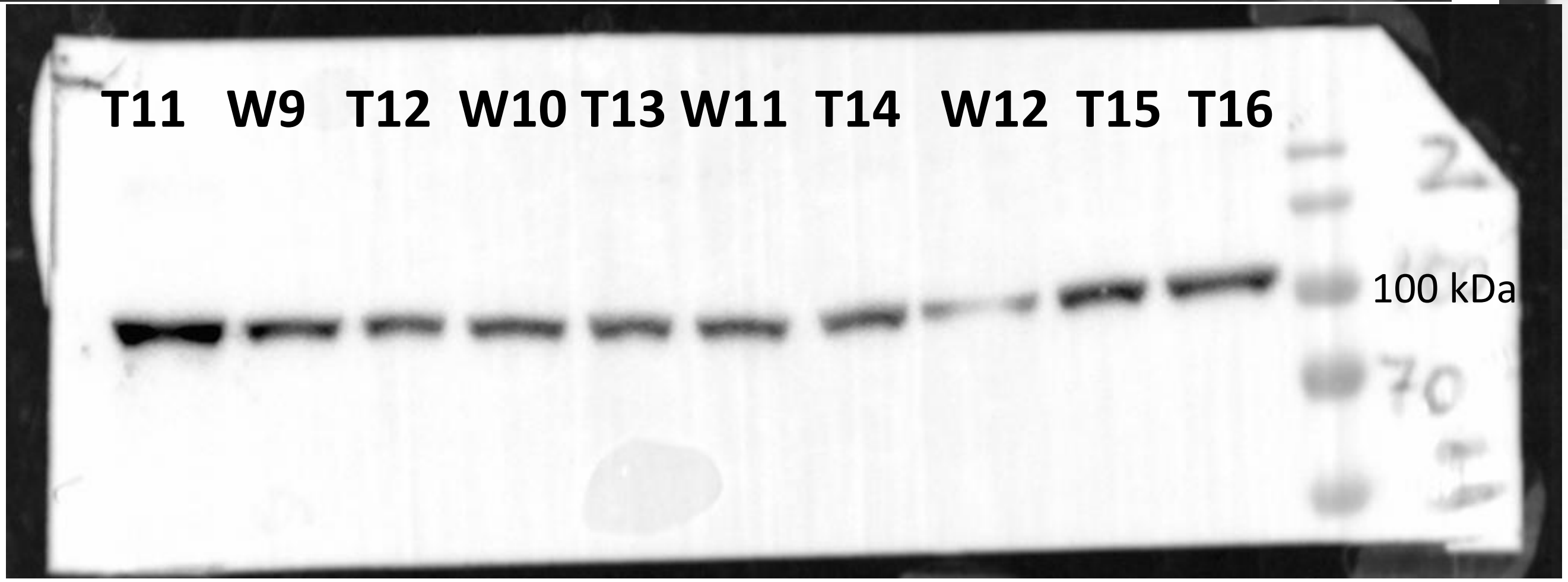

GAPDH (to INSR)

37 kDa

T5 W5 T6 W6 T7 W7 T8 W8 T9 T10

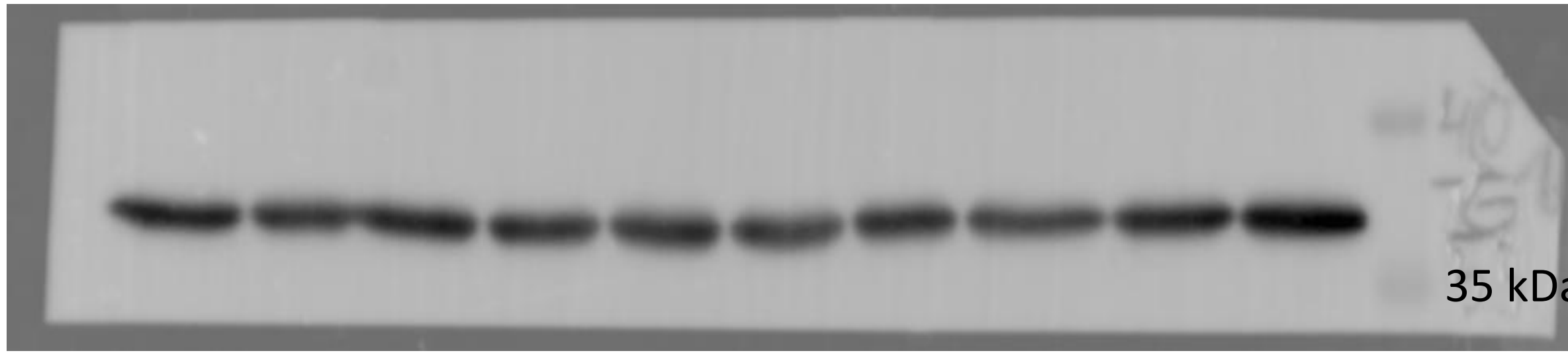

T11 W9 T12 W10 T13 W11 T14 W12 T15 T16

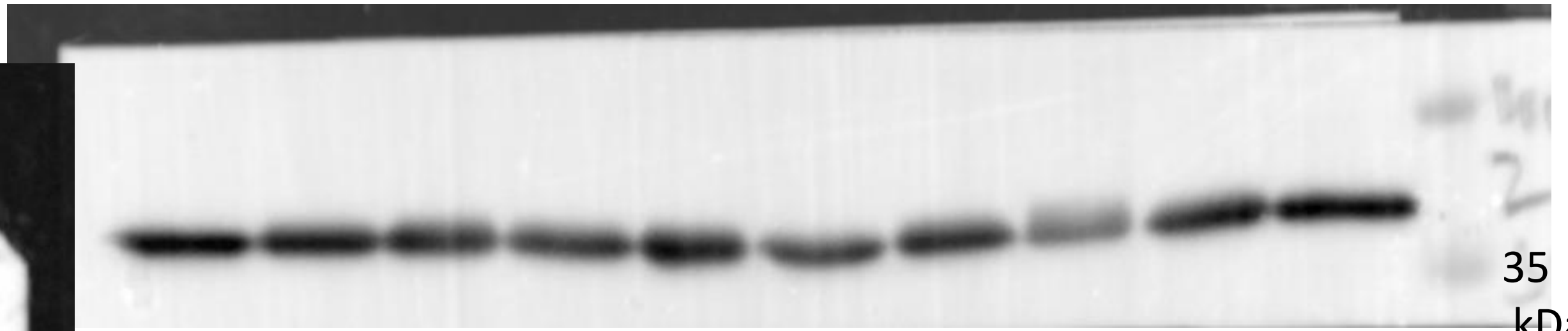

35 kDa

W = WT
T = PP2A-TG

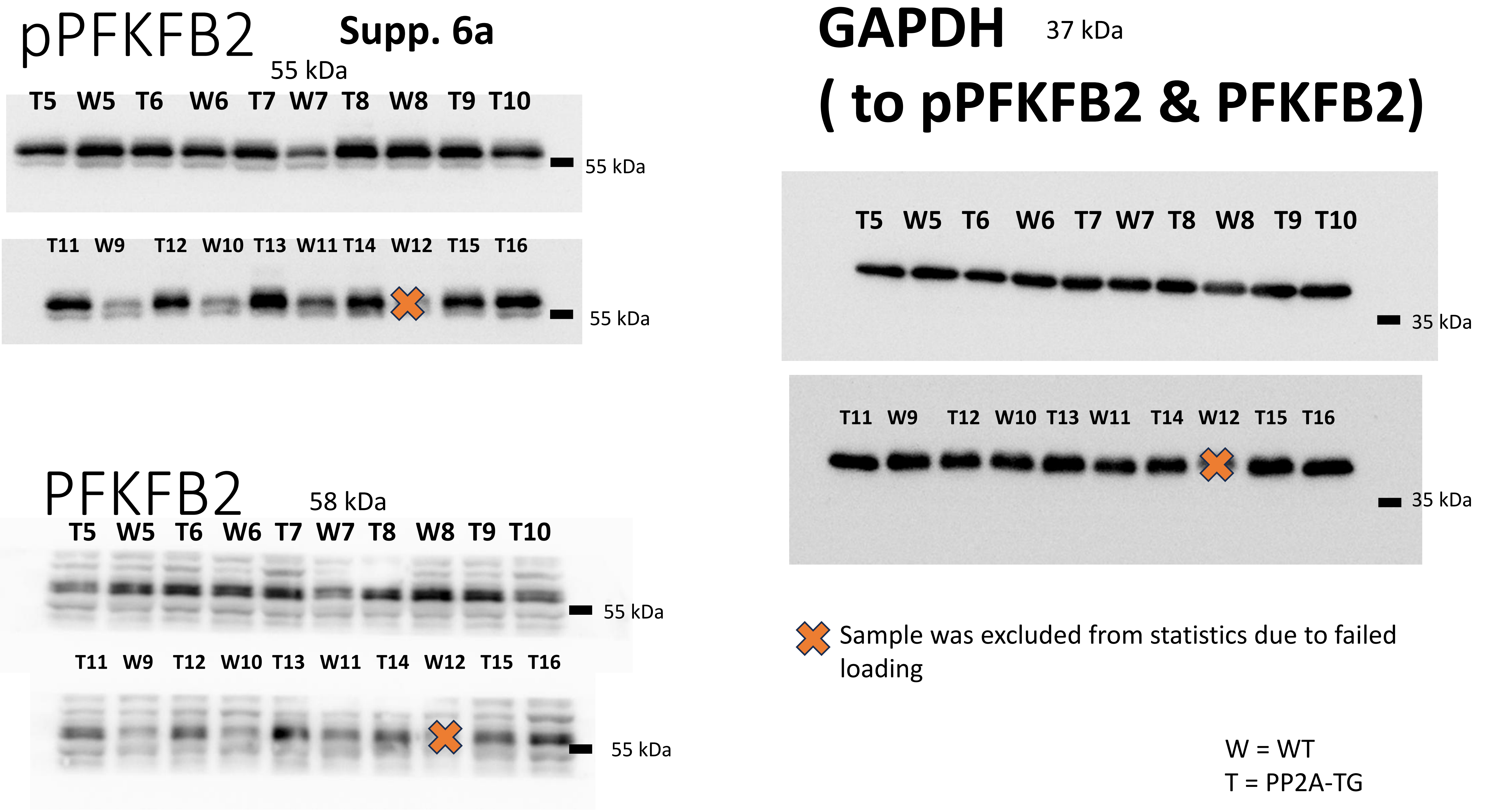
pPFKFB2
Supp. 6a
55 kDa
T5 W5 T6 W6 T7 W7 T8 W8 T9 T10
55 kDa
T11 W9 T12 W10 T13 W11 T14 W12 T15 T16
55 kDa
PFKFB2
58 kDa
T5 W5 T6 W6 T7 W7 T8 W8 T9 T10
55 kDa
T11 W9 T12 W10 T13 W11 T14 W12 T15 T16
55 kDa
GAPDH
37 kDa
(to pPFKFB2 & PFKFB2)
T5 W5 T6 W6 T7 W7 T8 W8 T9 T10
35 kDa
T11 W9 T12 W10 T13 W11 T14 W12 T15 T16
35 kDa
Sample was excluded from statistics due to failed loading
W = WT
T = PP2A-TG

Supp. 6b -marker

pPFKFB2 Ser483
55 kDa

T5 W5 T6 W6 T7 W7 T8 W8 T9 T10

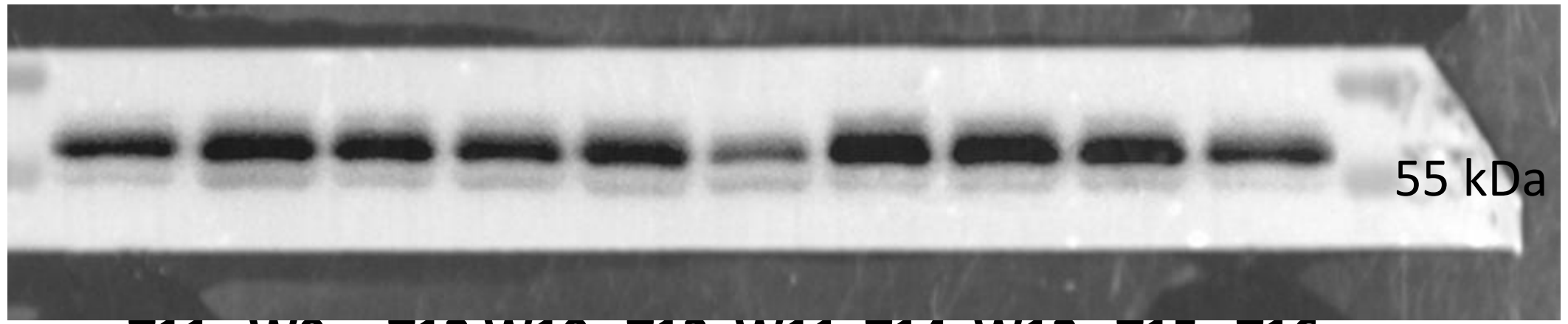

T11 W9 T12 W10 T13 W11 T14 W12 T15 T16

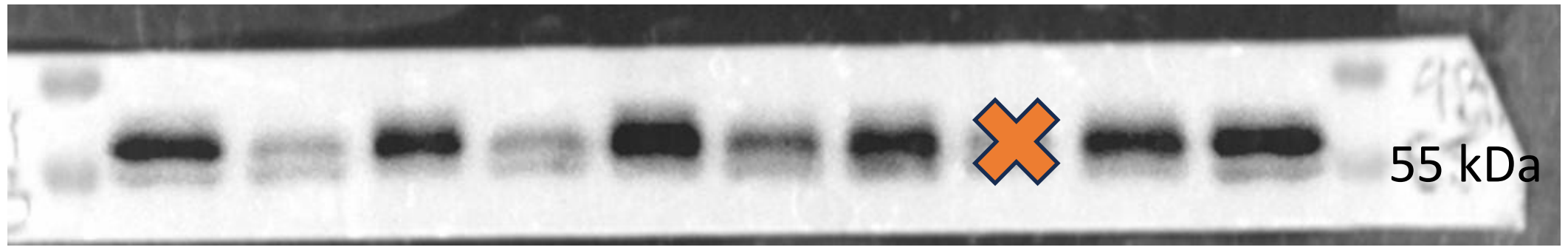

PFKFB2 58 kDa

T5 W5 T6 W6 T7 W7 T8 W8 T9 T10

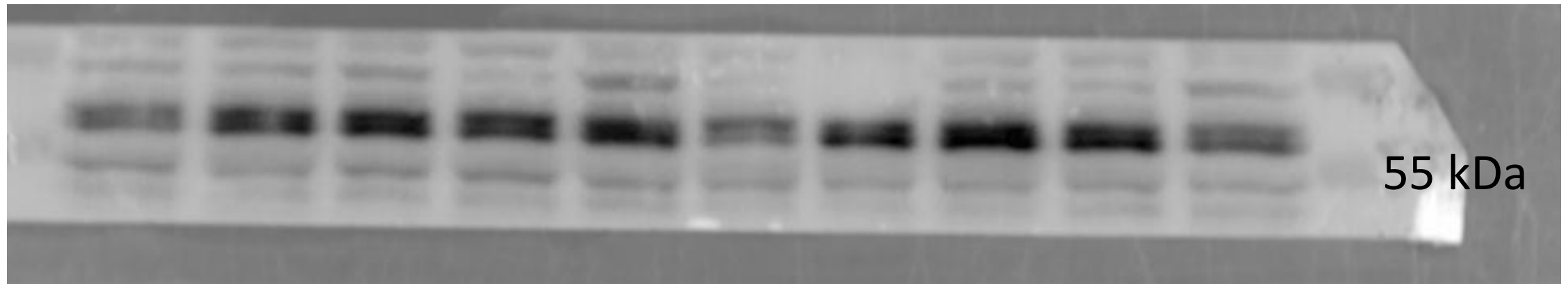

T11 W9 T12 W10 T13 W11 T14 W12 T15 T16

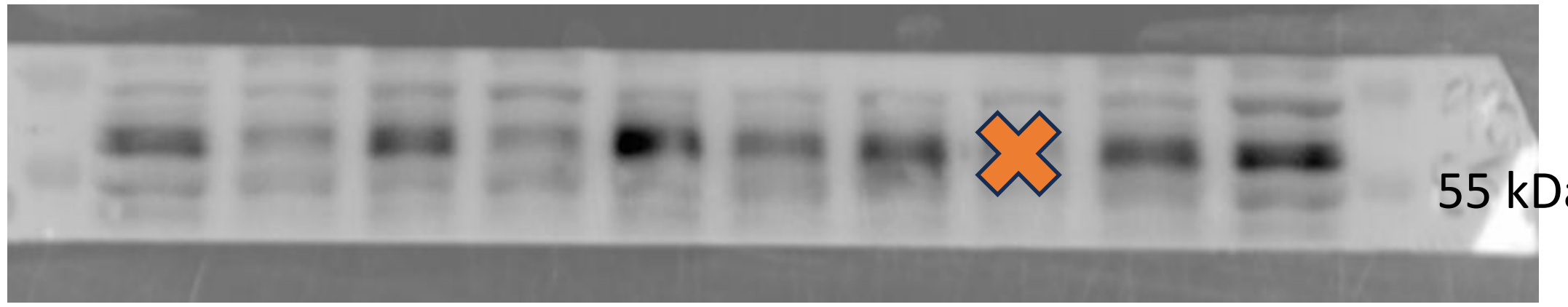

GAPDH 37 kDa
(to pPFKFB2 & PFKFB2)

T5 W5 T6 W6 T7 W7 T8 W8 T9 T10

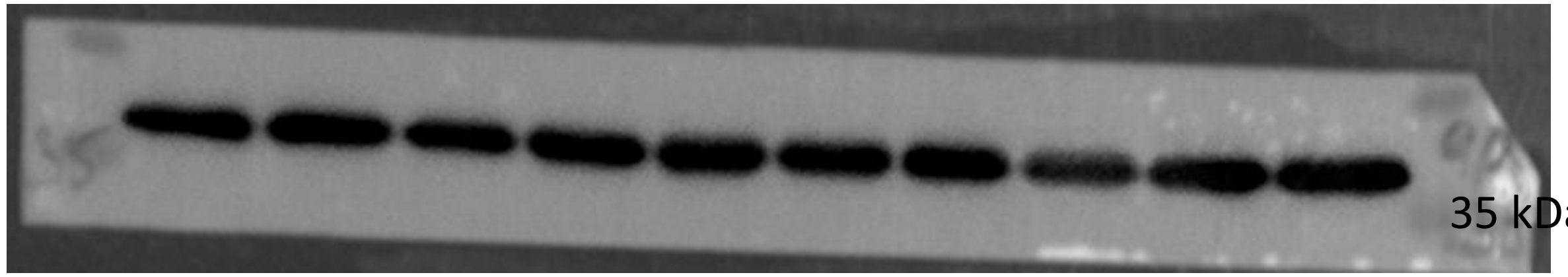

35 kDa

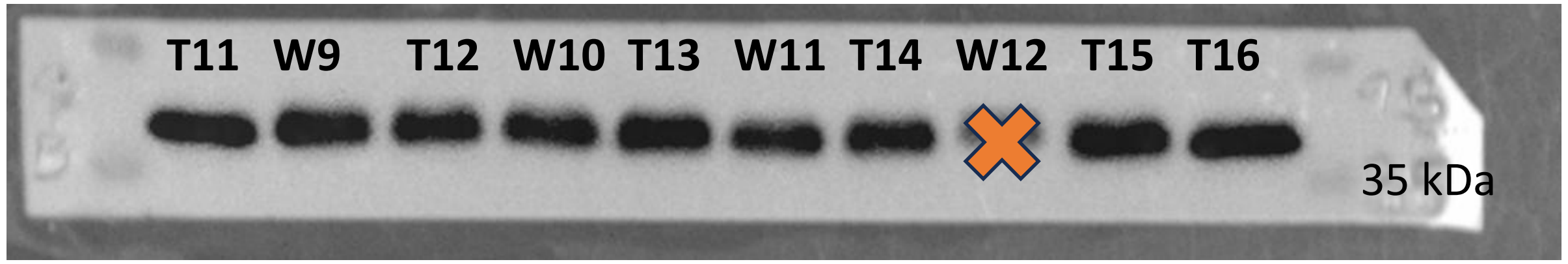

W = WT
T = PP2A-TG

Supp. 7a

PI3K 100 kDa observed 105 kDa

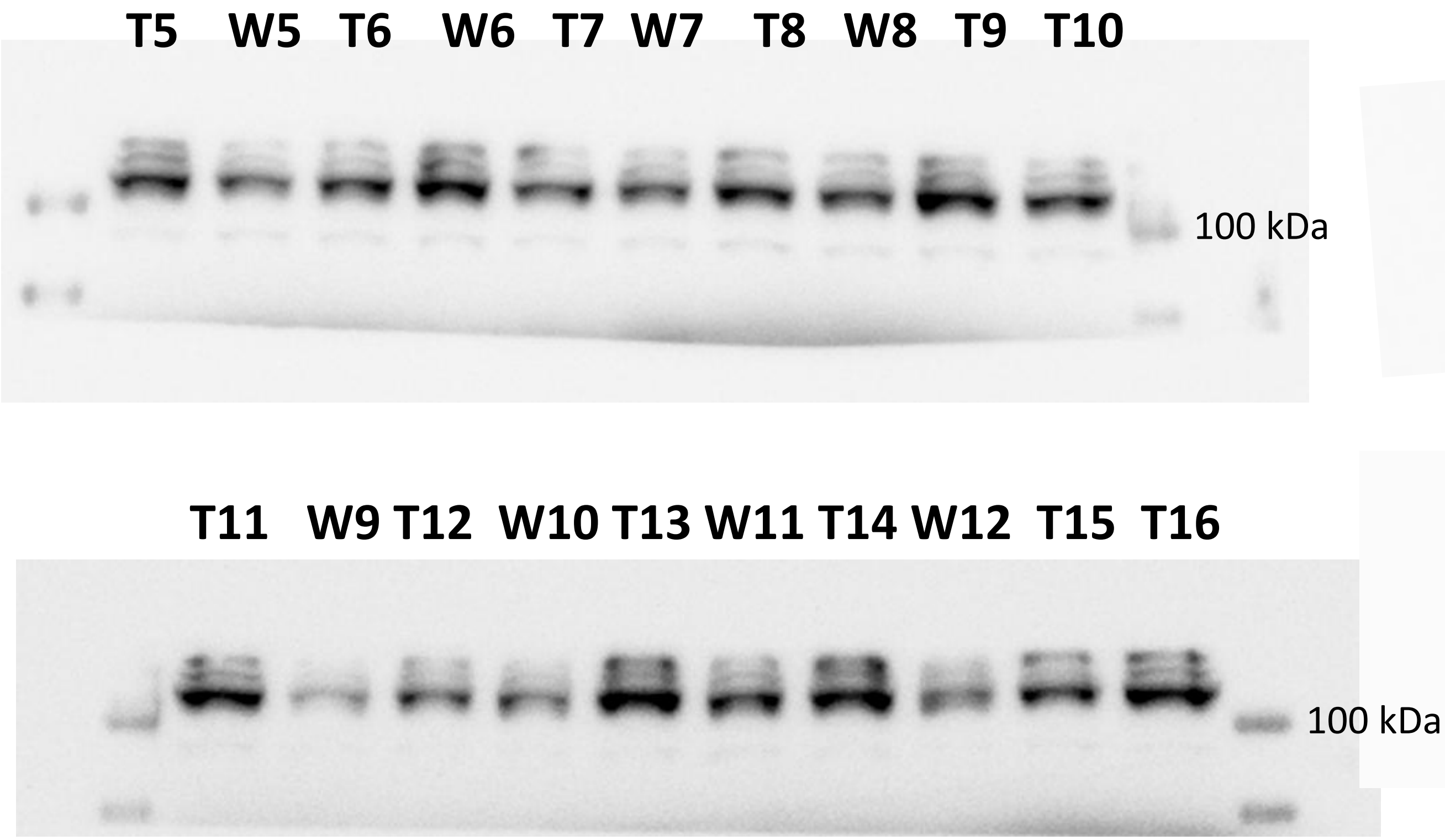

GAPDH (to PI3K) 37 kDa

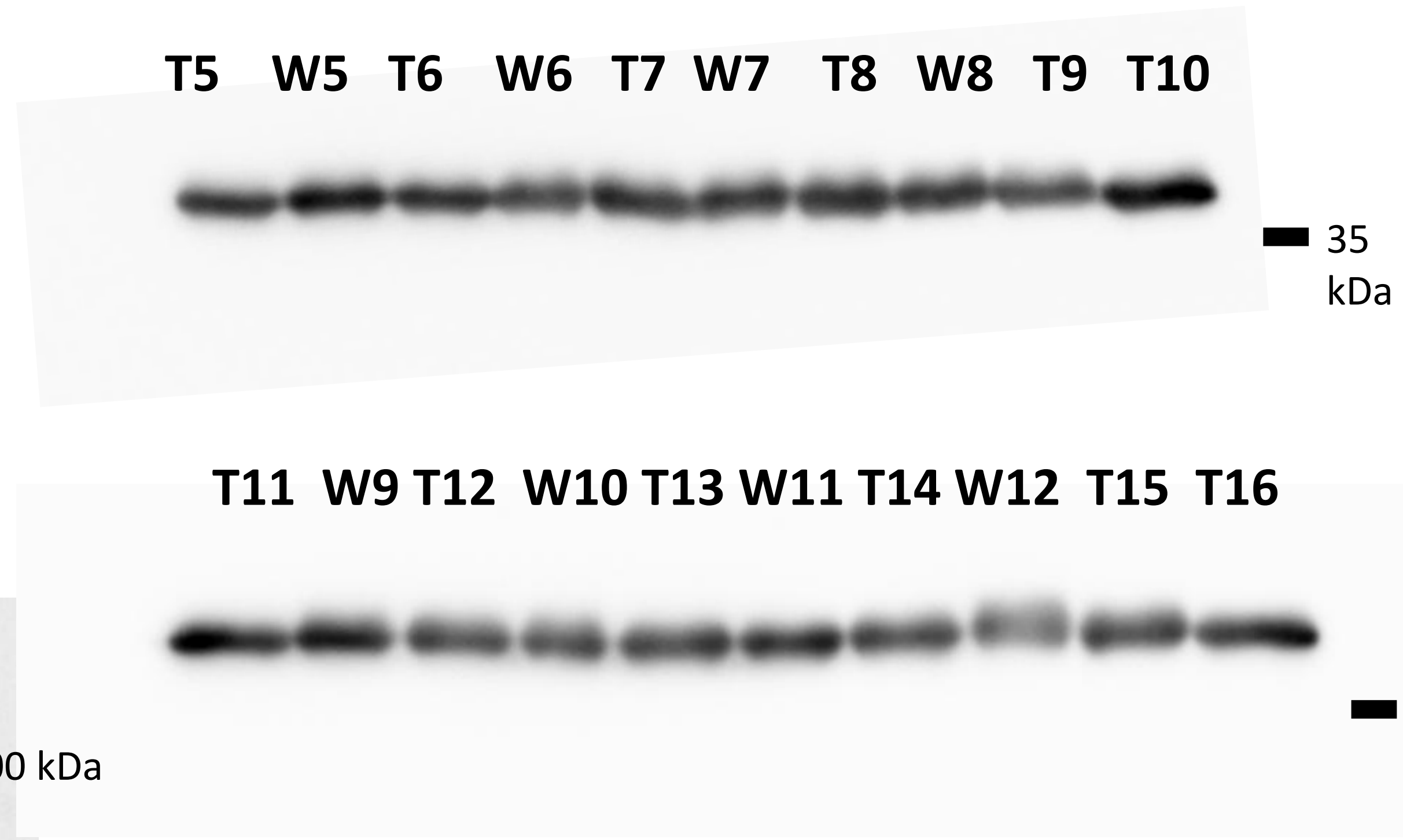

W = WT

T = PP2A-TG

PI3K 100 kDa observed 105 kDa

Supp. 7b- marker

T5 W5 T6 W6 T7 W7 T8 W8 T9 T10

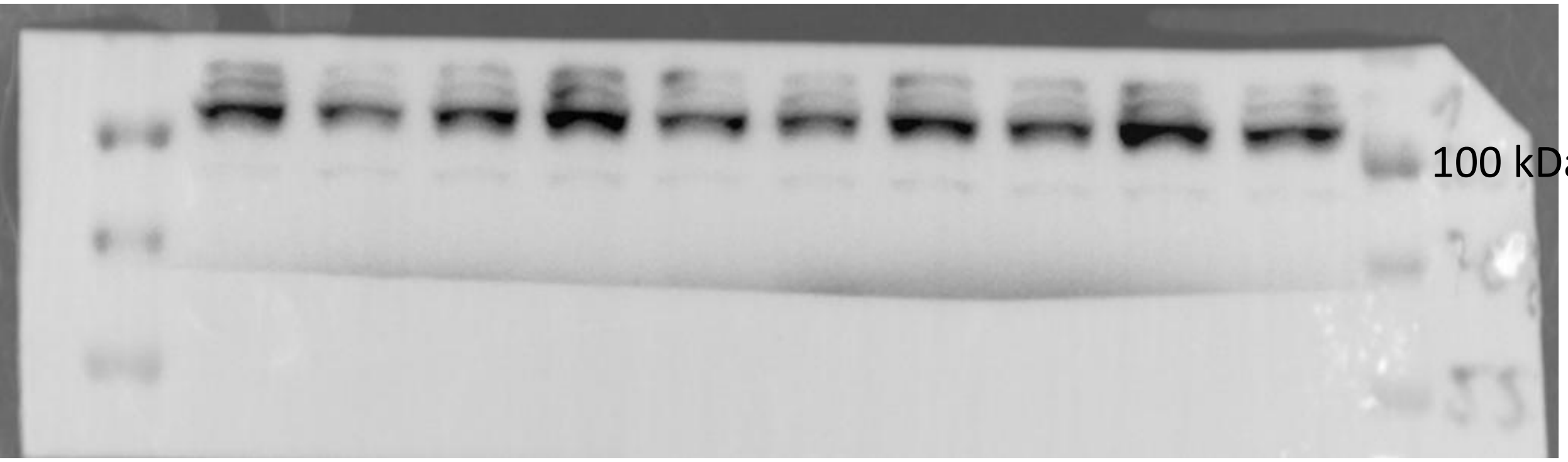

T11 W9 T12 W10 T13 W11 T14 W12 T15 T16

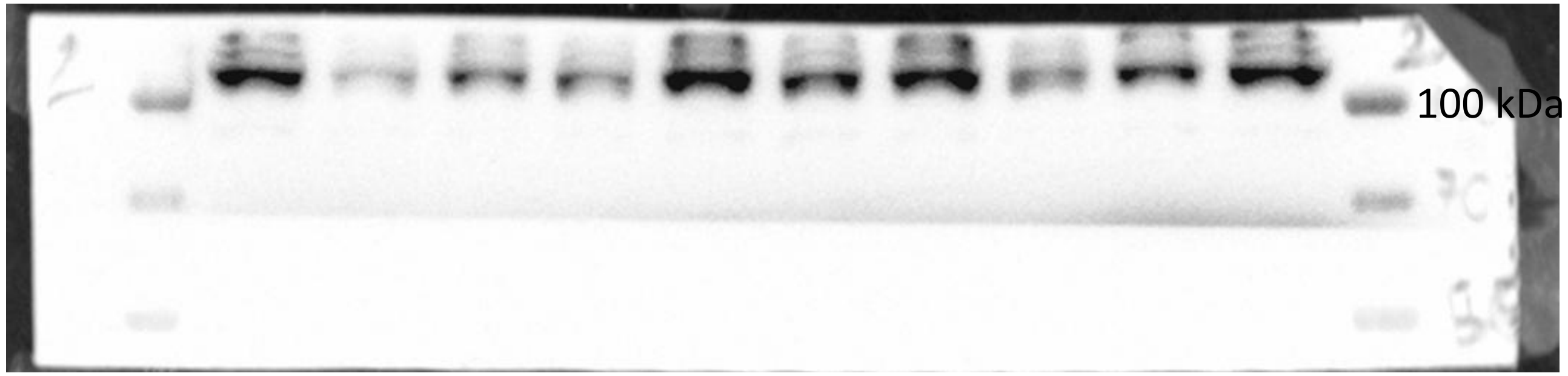

GAPDH (to PI3K) 37 kDa

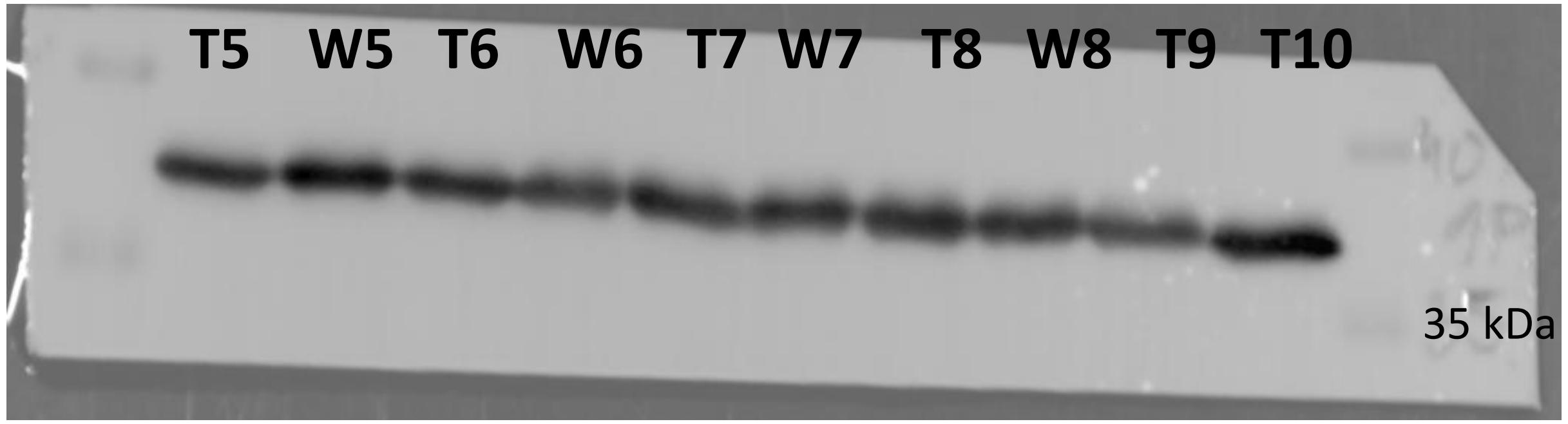

T11 W9 T12 W10 T13 W11 T14 W12 T15 T16

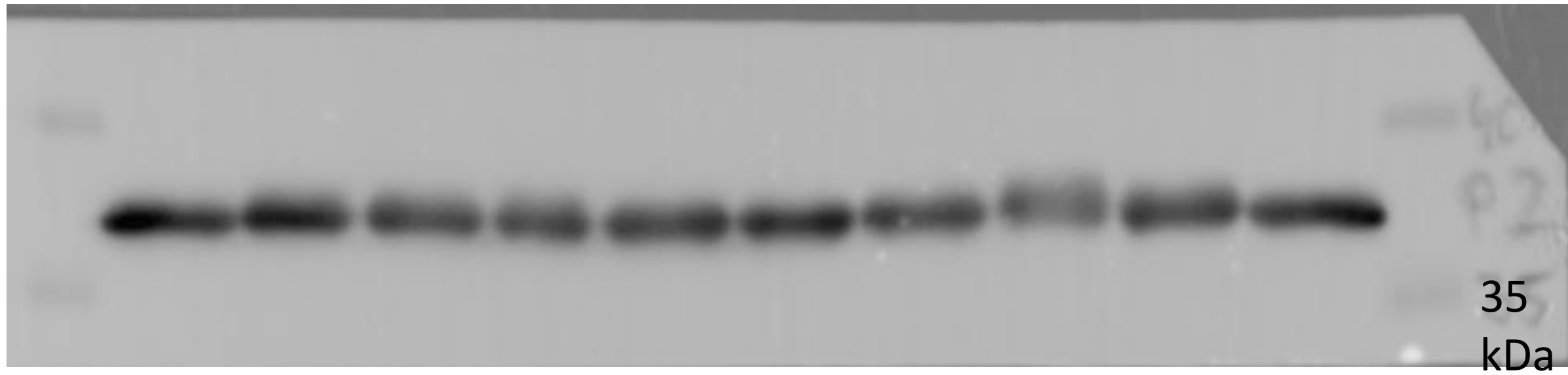

W = WT

T = PP2A-TG

Supp. 8a

PYGM 97 kDa observed 105 kDa

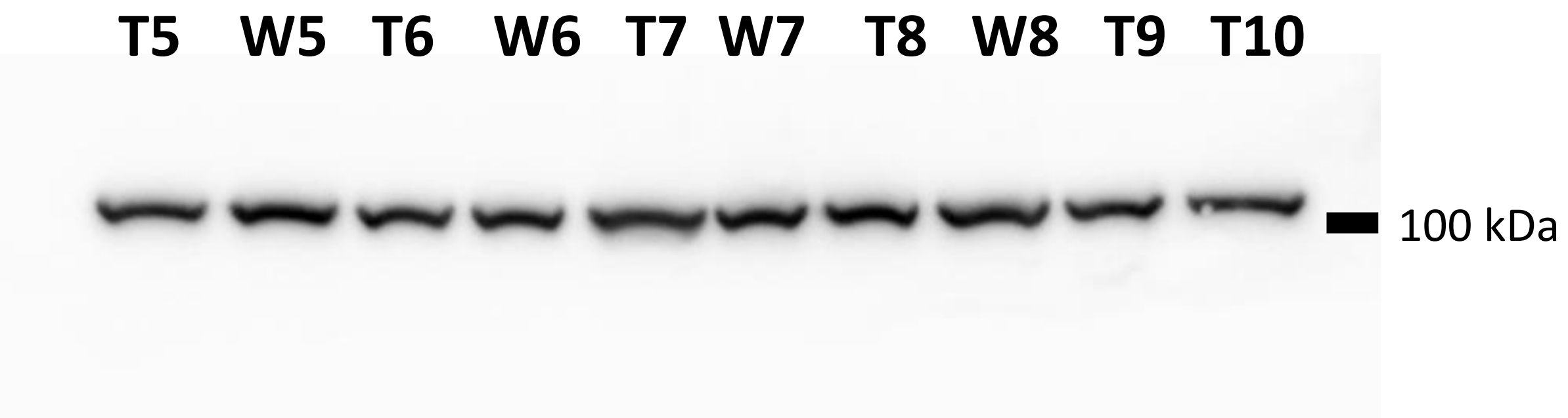

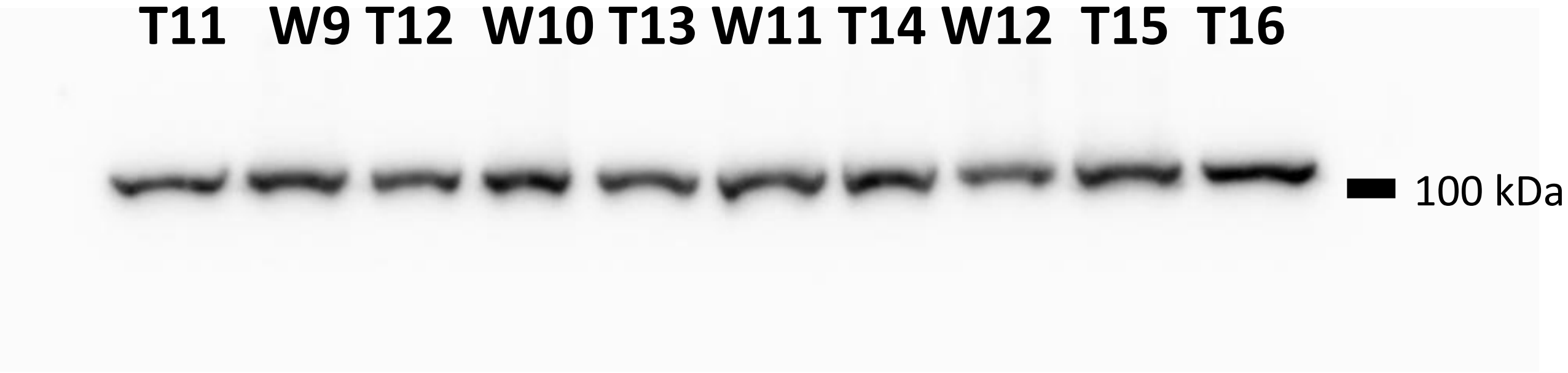

GAPDH (to PYGM) 37 kDa

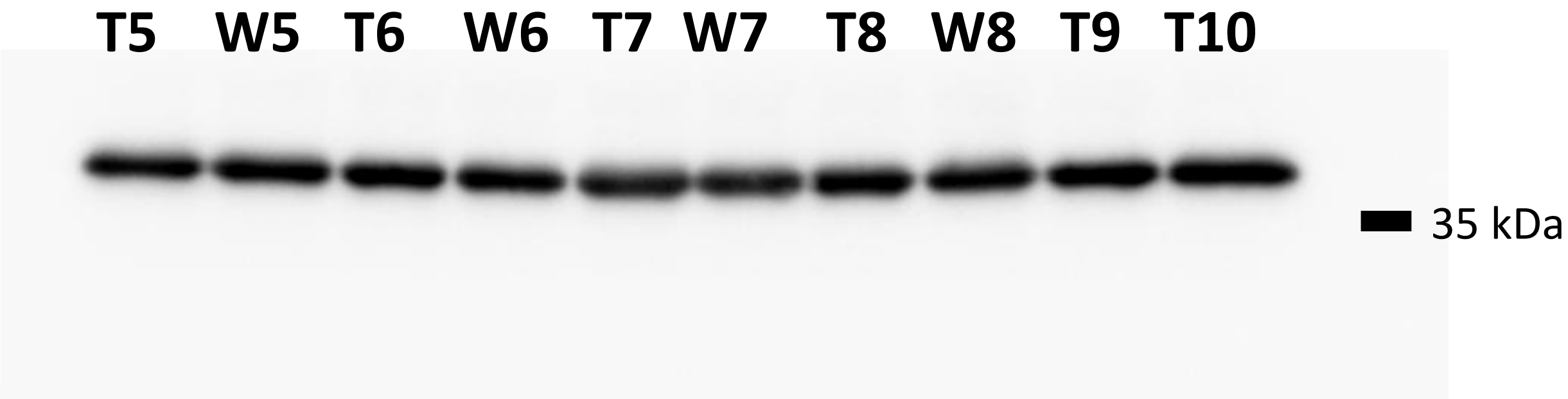

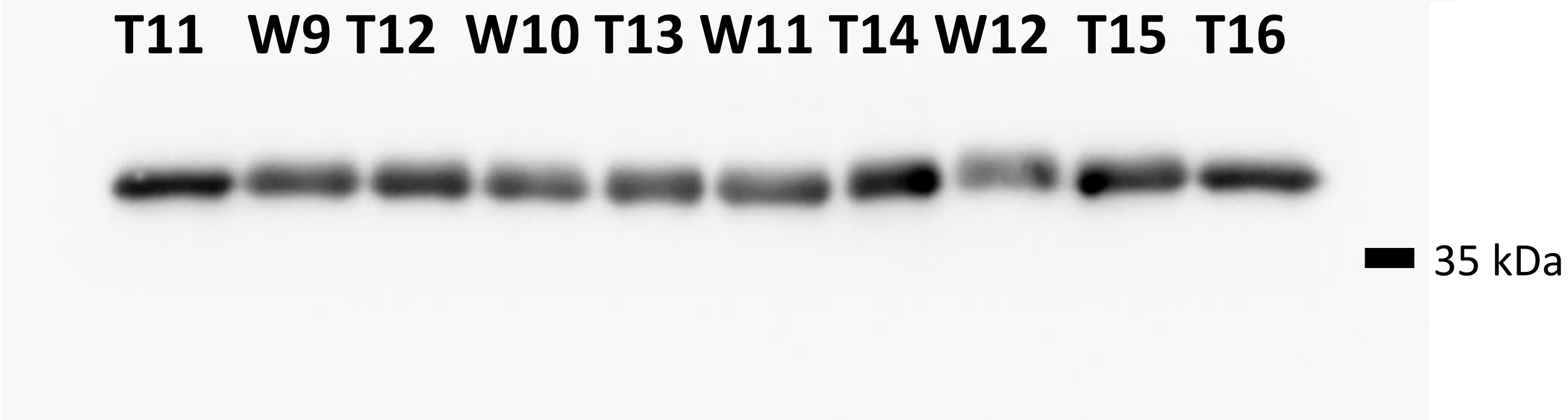

W = WT
T = PP2A-TG

Supp. 8b - marker

PYGM 97 kDa

T5 W5 T6 W6 T7 W7 T8 W8 T9 T10

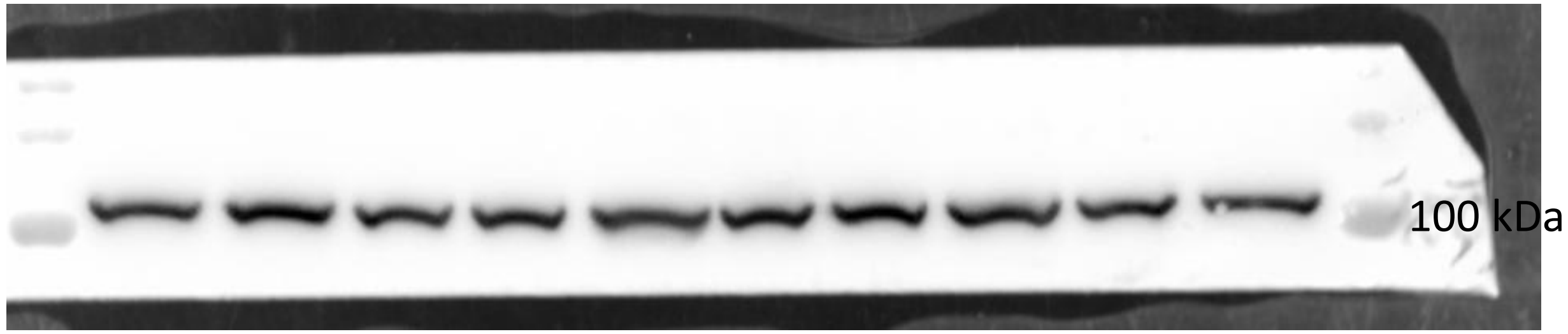

T11 W9 T12 W10 T13 W11 T14 W12 T15 T16

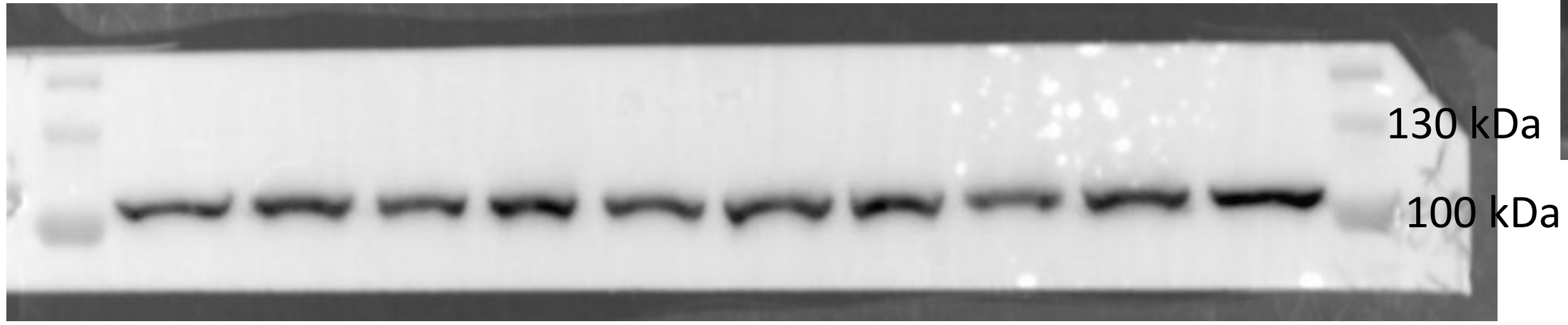

GAPDH (to PYGM) 37 kDa

T5 W5 T6 W6 T7 W7 T8 W8 T9 T10

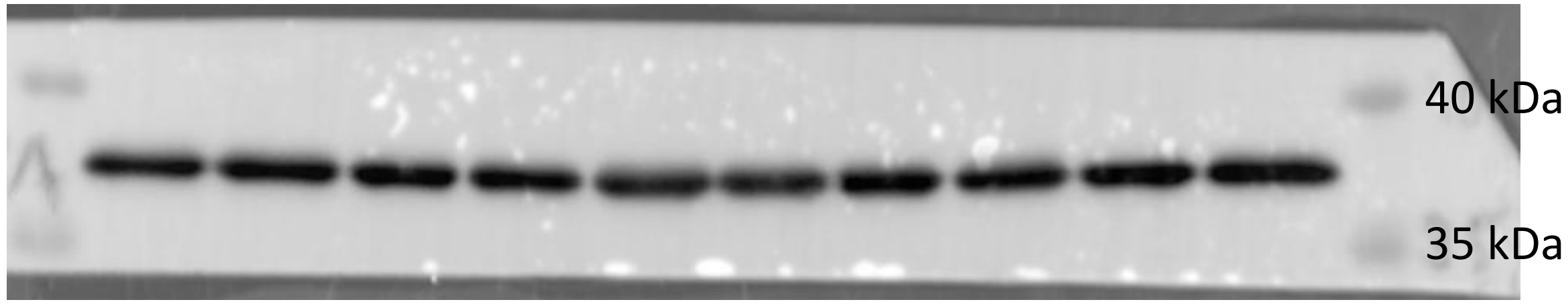

T11 W9 T12 W10 T13 W11 T14 W12 T15 T16

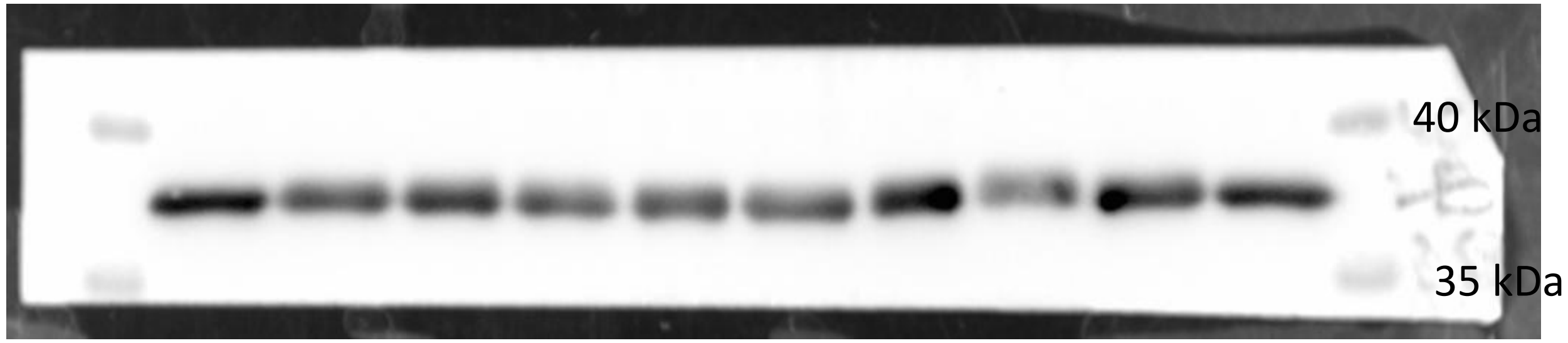

W = WT

T = PP2A-TG
